# Supplementary figures and images for: Structure of the Pseudomonas aeruginosa PAO1 Type IV pilus
Source: PLoS Pathog. 2024 Dec 12;20(12):e1012773. doi: 10.1371/journal.ppat.1012773 (PMC11670995; doi:10.1371/journal.ppat.1012773)

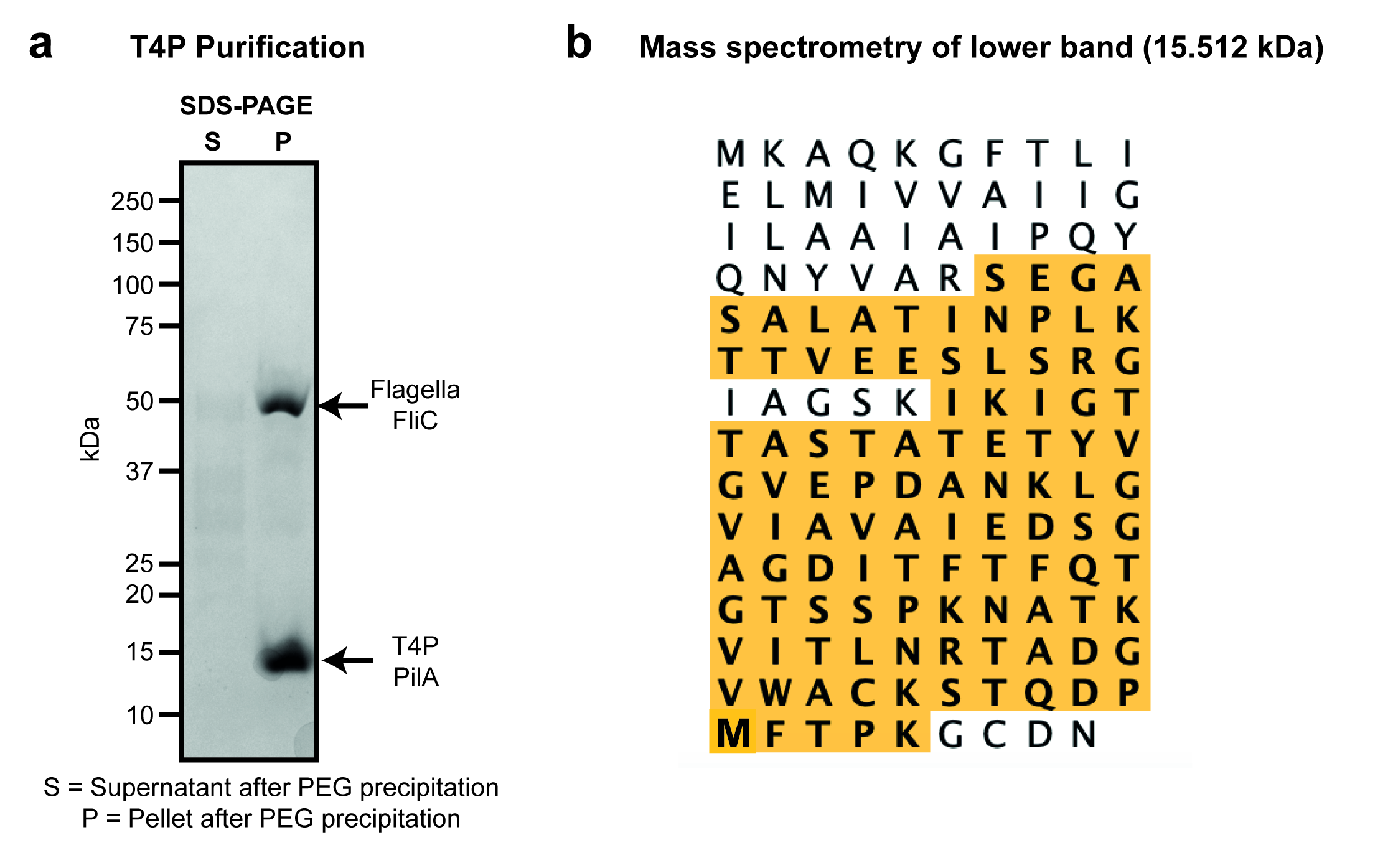

Supplement: S1 Fig — a SDS-PAGE analysis of the final specimen after PEG precipitation (see Methods). Two bands, corresponding to FliC (flagella) and T4P PilA, are present at the expected molecular weights. The positions of molecular weight markers are shown on the left. b Peptide-fingerprinting mass spectrometry result of the T4P PilA band. The detected peptides are highlighted in the sequence, confirming the presence of T4P PilA in our sample. (TIF) [file ppat.1012773.s001.tif]

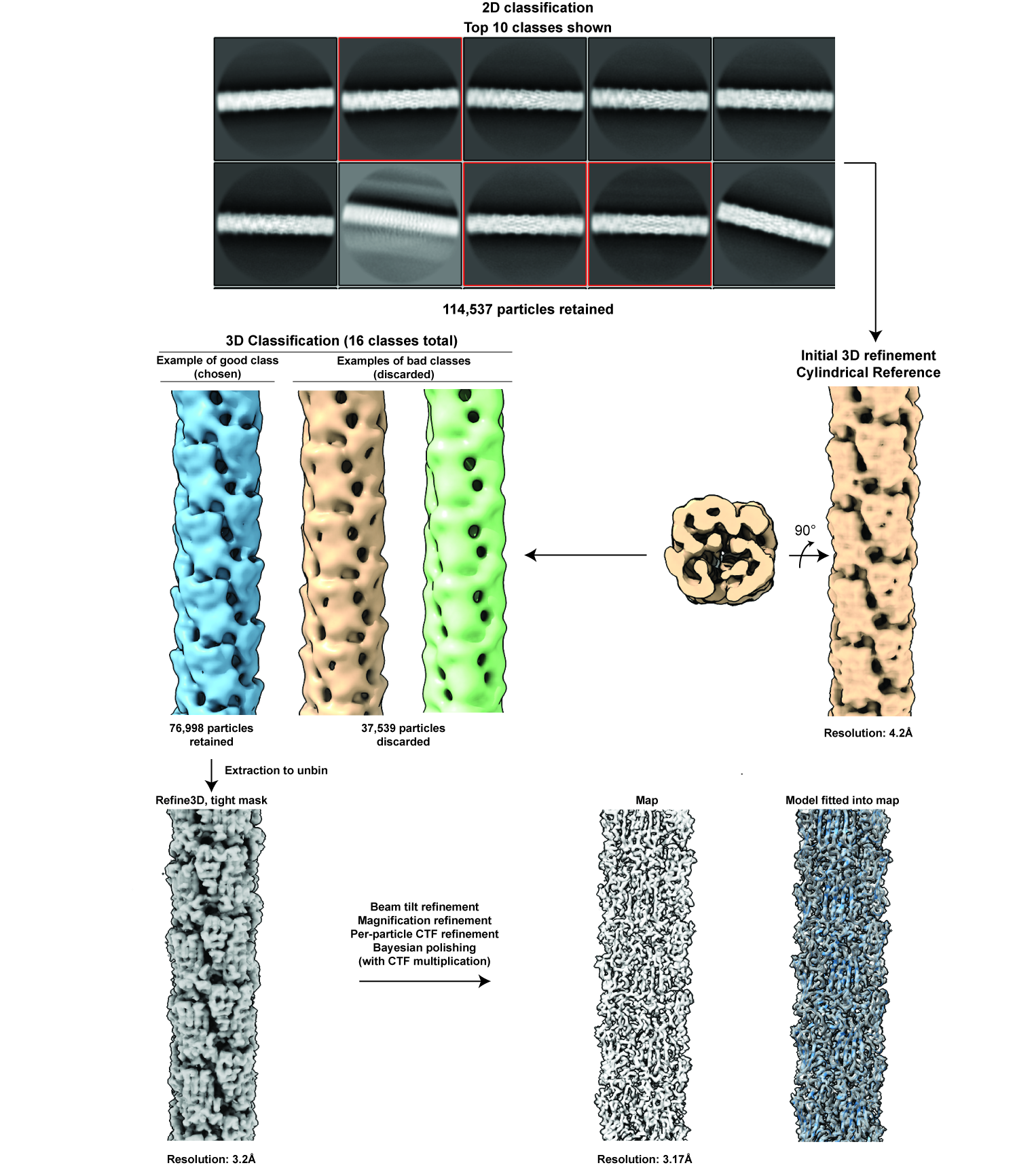

Supplement: S2 Fig — After particle picking and 2D classification (top 10 populated classes shown), the three best classes (114,537 particles, marked in red) were selected and used for initial 3D refinement yielding a 4.2 Å-resolution pilus structure. Following that, 3D classification was performed resulting in a final set of 76,998 particles, which were used for further 3D refinement. Further refinements and polishing were performed, resulting in the final 3.17 Å-resolution map. (TIF) [file ppat.1012773.s002.tif]

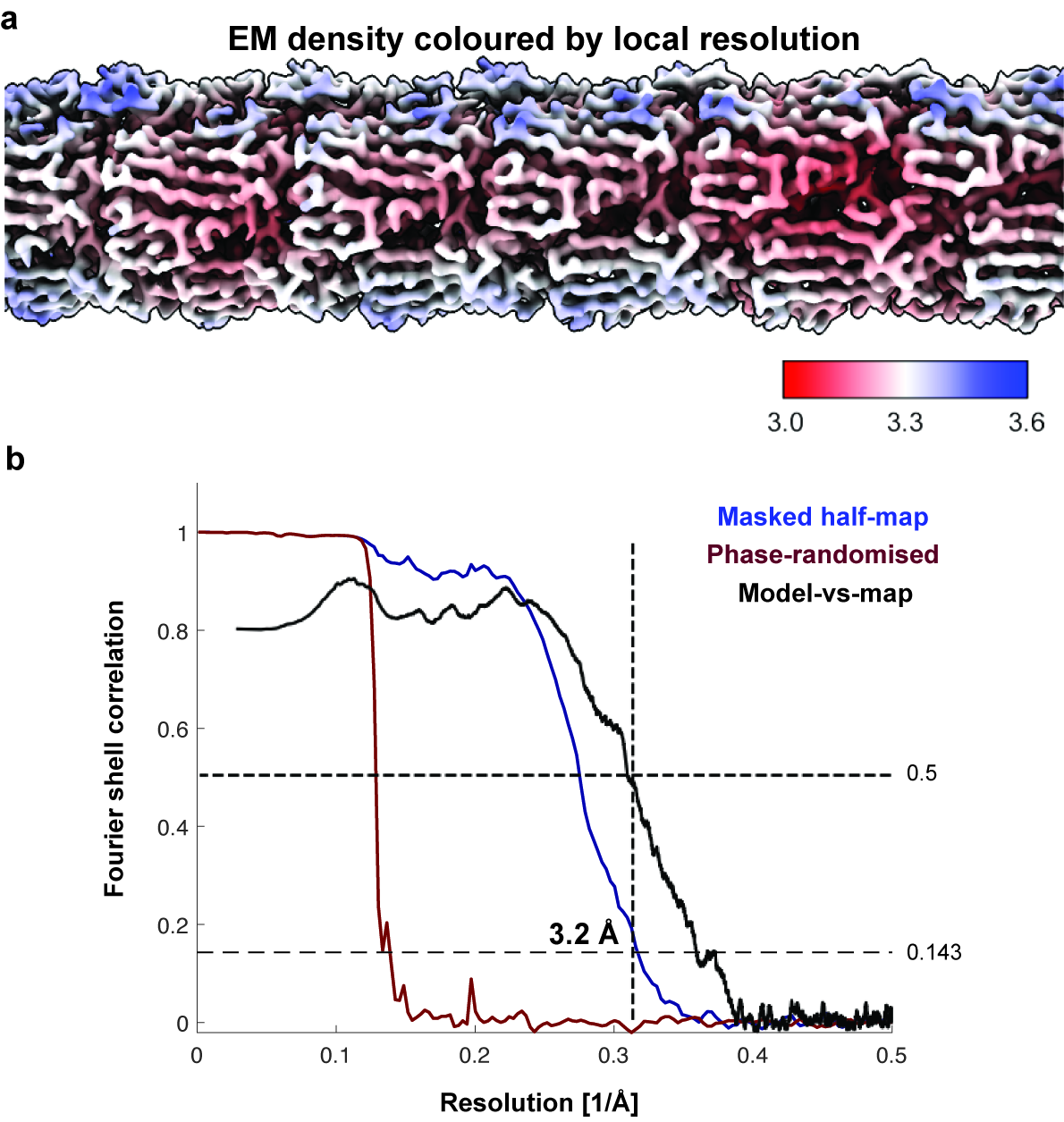

Supplement: S3 Fig — a Cryo-EM density map coloured by local resolution (calculated in RELION 4.0). The slightly lower local resolution on the outside of the T4P is indicative of a higher degree of structural flexibility of the β-sheet domain compared to the α-helical part of the pilin. b Solvent-corrected masked half-map FSC (blue, gold-standard), phase-randomised half-map FSC (red), and model-vs-map FSC (black). (TIF) [file ppat.1012773.s003.tif]

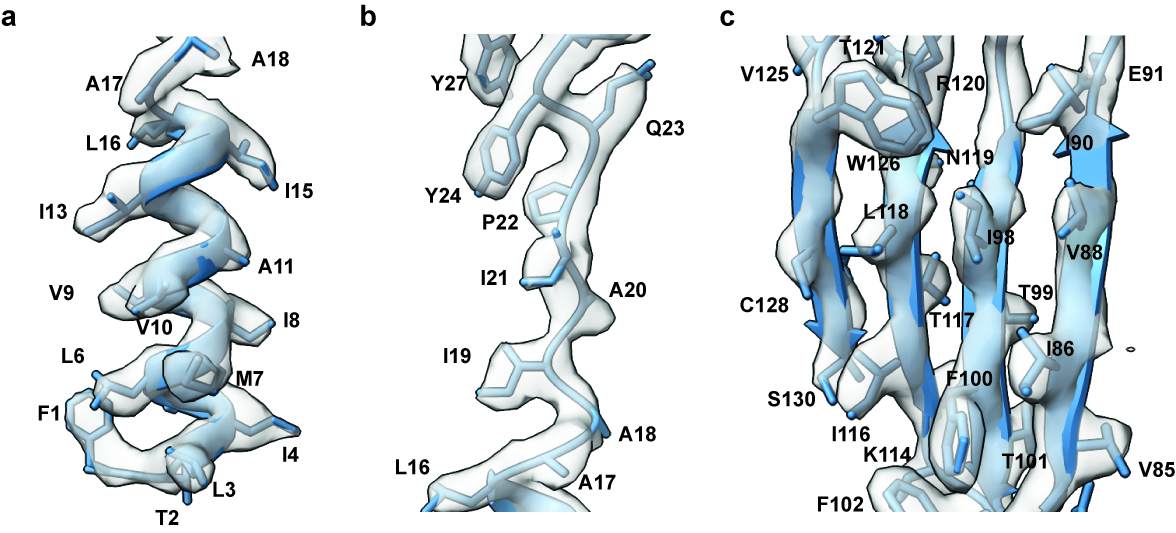

Supplement: S4 Fig — a-c Models of the lower part of the N-terminal helix (residues 1–16, a), the ‘melted’ linker segment (residues 17–24, b) and the β-sheet (residues 85–130, c) fitted into the cryo-EM density. The side chains are clearly resolved in the map. (TIF) [file ppat.1012773.s004.tif]

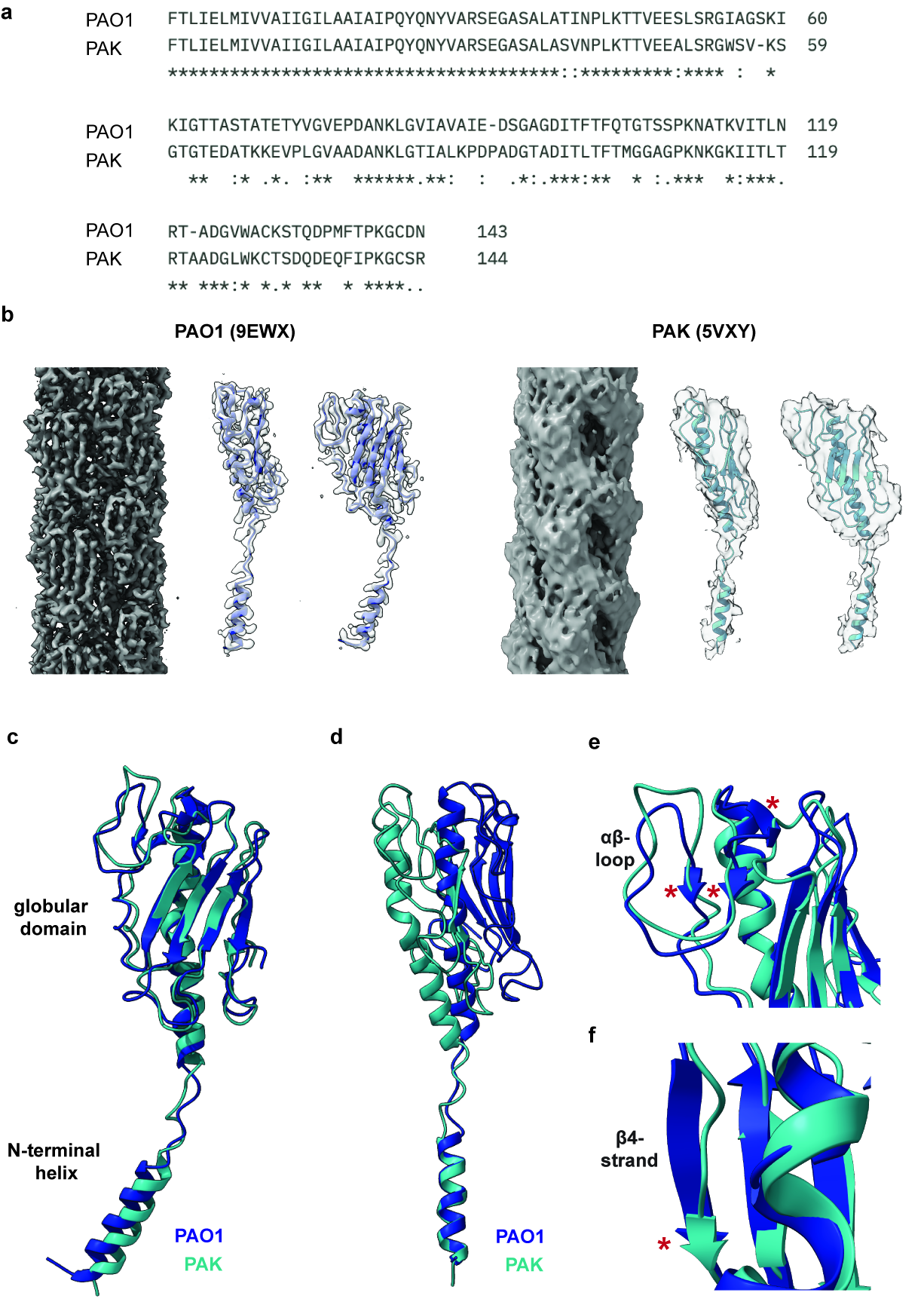

Supplement: S5 Fig — a Sequence alignment [90] of P. aeruginosa pilA from strains PAO1 and PAK. b EM density map of the PAO1 T4P reported in this paper (EMD-50025), side view and front view of the EM density around one pilin subunit (PDB: 9EWX, this study) and EM density map of the PAK T4P reported in a previous study [26] (EMD-8740), along with side view and front view of the EM density around one pilin subunit (PDB: 5VXY). c-f Alignment of the pilin structures of PAO1 (dark blue) and PAK (teal), based on the globular domain (c, e, f) or the N-terminal helix (d), shows secondary structure differences, highlighting the relevance of a high-resolution map featuring side chain densities. Specifically, there is a difference in orientation between the N-terminal helix and the globular domain (c, d). In the globular domain, PAO1 features additional small secondary structure elements within the αβ-loop, while the loop between beta strands β1 and β2 is extended in PAK (red asterisks) (e). There are also differences in beta strand length between the two structures (red asterisk) (f). (TIF) [file ppat.1012773.s005.tif]

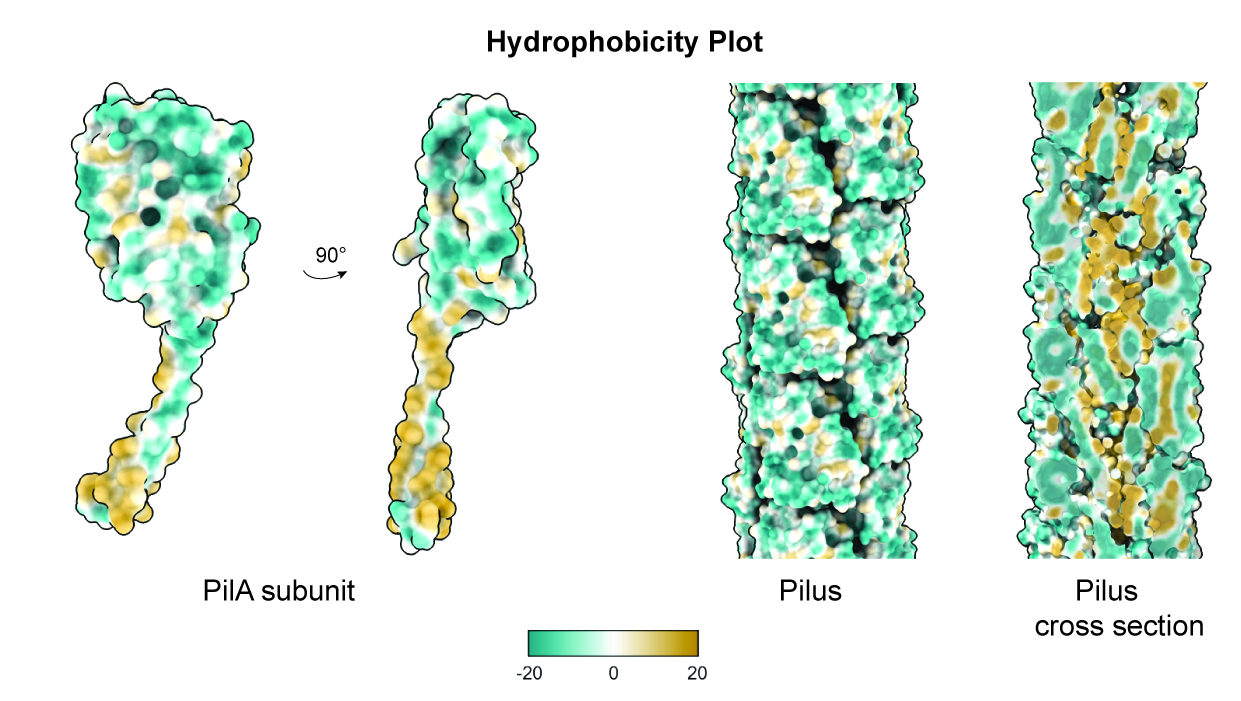

Supplement: S6 Fig — Hydrophobicity of the P. aeruginosa PilA subunit and the fully assembled T4P. The subunit is shown in two orientations related to each other by a 90° rotation (left). For the assembled pilus (right), both the outer surface and a cross section through the centre are shown. The models have been coloured by molecular lipophilicity potential using the mlp function in ChimeraX [86]. (TIF) [file ppat.1012773.s006.tif]

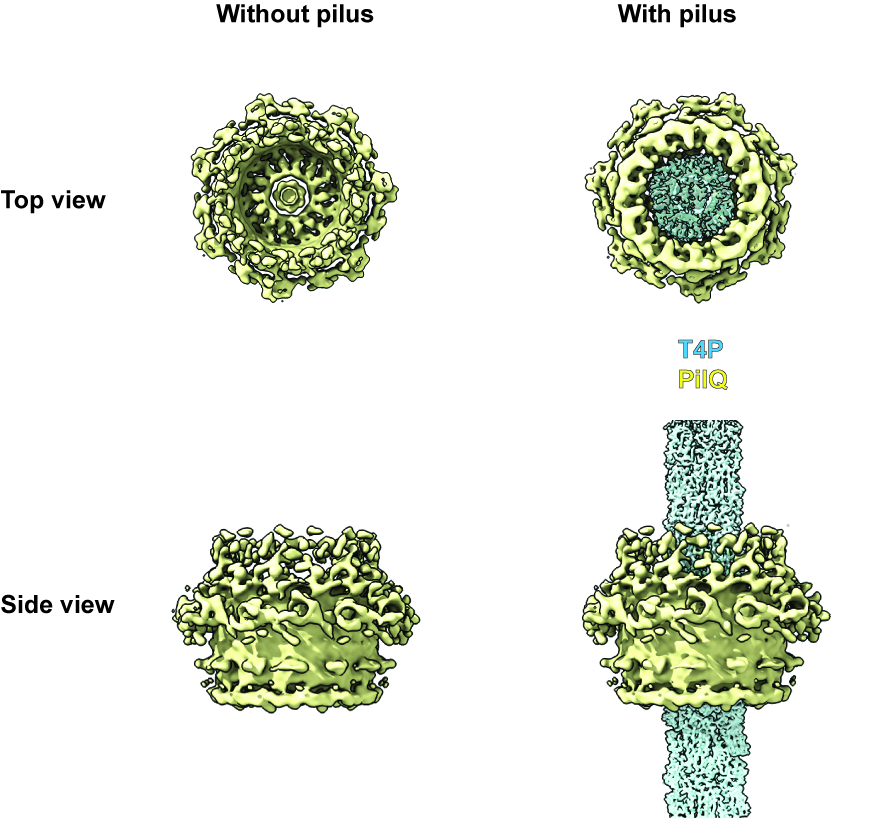

Supplement: S7 Fig — The secretin features a density in the lumen (top left) corresponding to the central gate, which needs to be displaced to accommodate the full pilus as described by Koo et al. [35]. The PAO1 T4P density fits well into the PilQ lumen. (TIF) [file ppat.1012773.s007.tif]

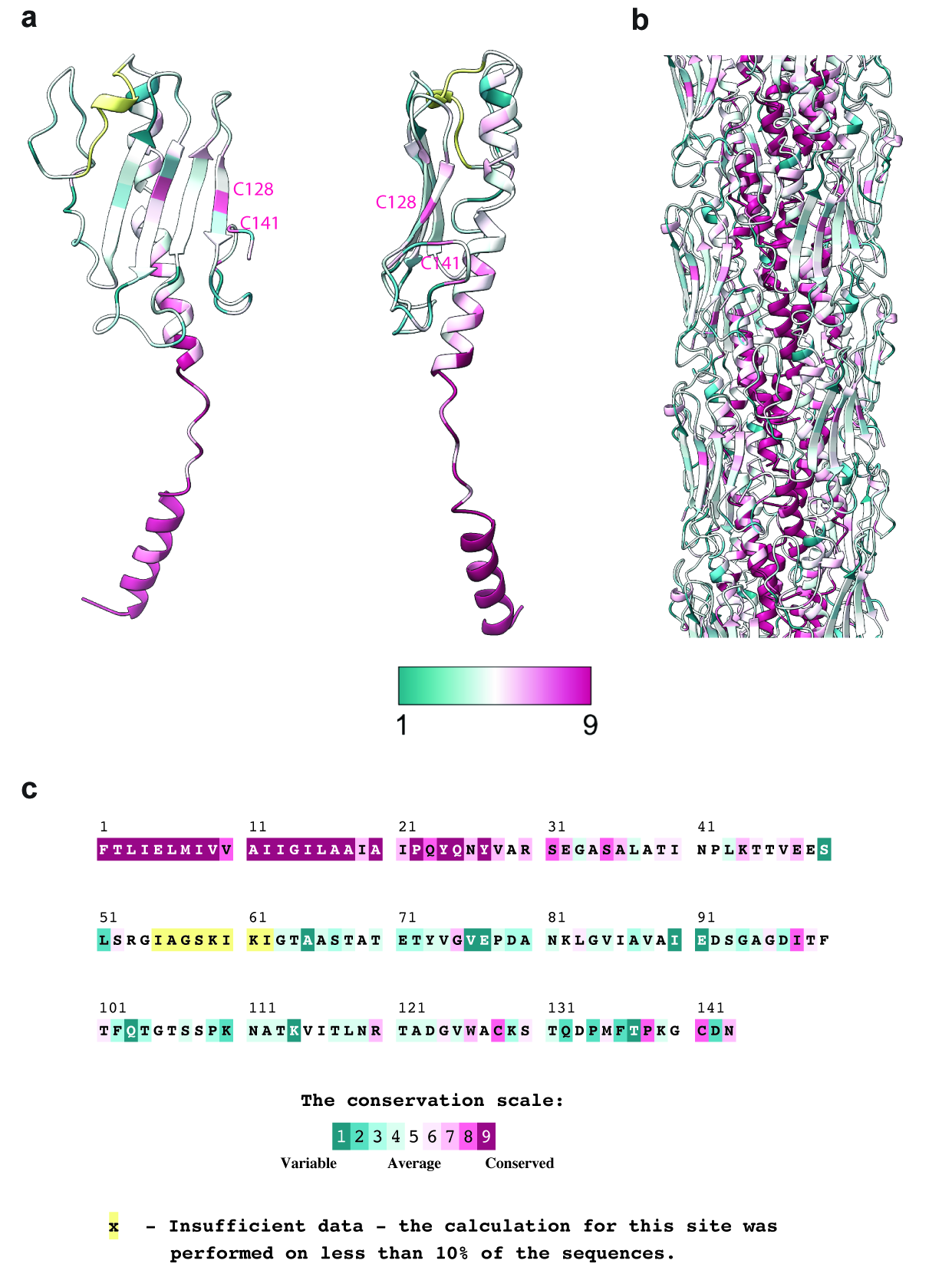

Supplement: S8 Fig — a Pilin structure coloured by ConSurf conservation score, where low numerical values (green) indicate low degrees and high numerical values (red) indicate high degrees of conservation. The analysis reveals a high degree of conservation of the N-terminal helix. A disulfide bond near the C-terminus (cysteine residues marked in pink) is also a feature conserved across many T4P species. b Full pilus structure coloured by ConSurf conservation score. c Sequence alignment and conservation score of the pilin from ConSurf analysis. (TIF) [file ppat.1012773.s008.tif]

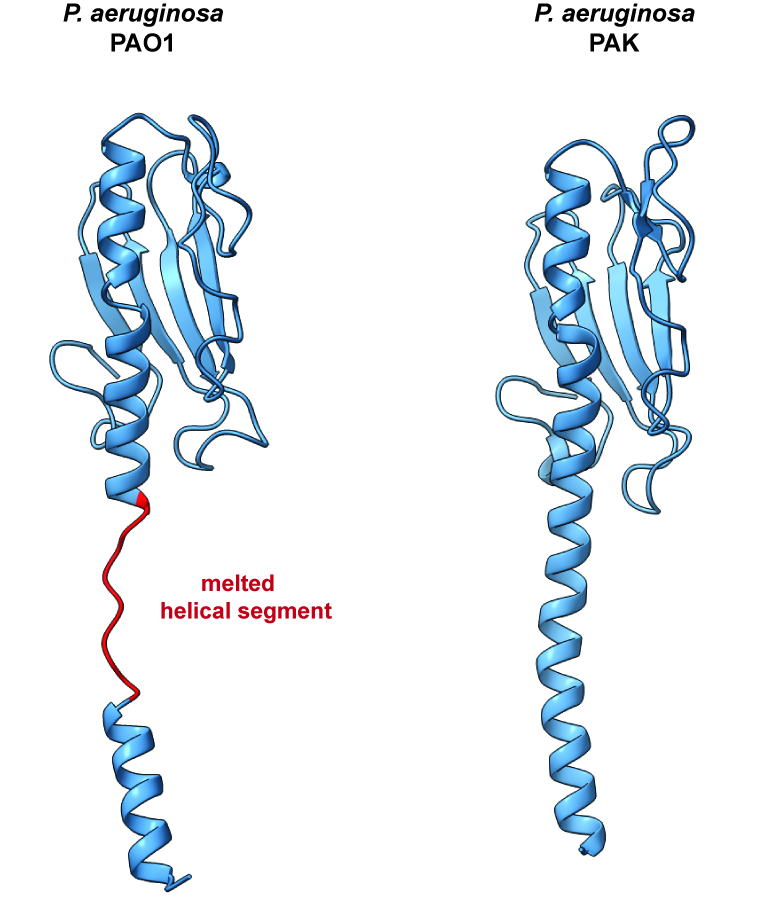

Supplement: S9 Fig — Side-by-side comparison of the PAO1 PilA (this study, left) with the PAK PilA (PDB: 1OQW, right) demonstrating the absence of the melted helical segment in the latter. (TIF) [file ppat.1012773.s009.tif]

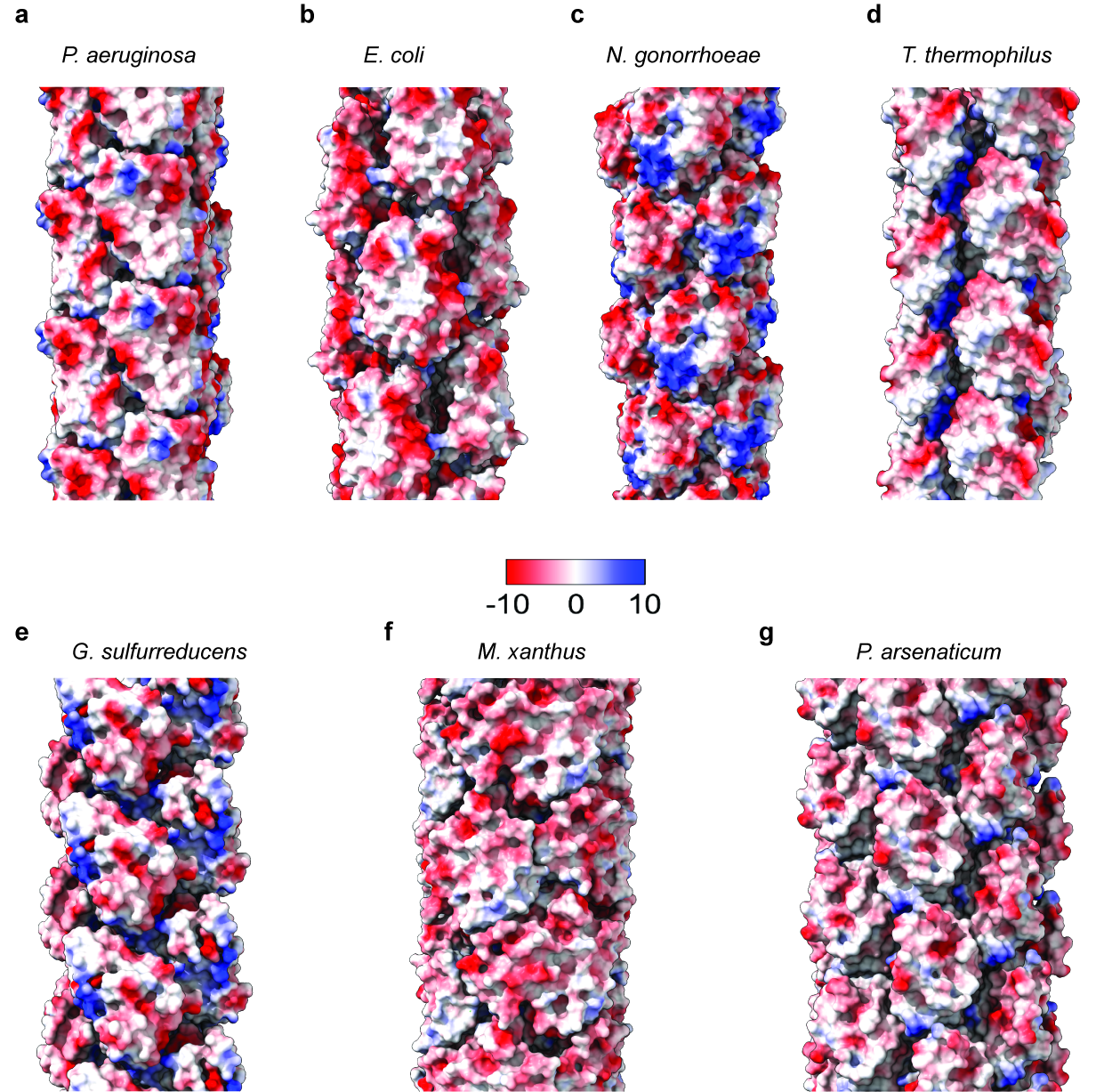

Supplement: S10 Fig — a-g Models of T4P structures from different organisms coloured by electrostatic surface potential using the coulombic function in ChimeraX [86]: P. aeruginosa PAO1 (this study, PDB: 9EWX) (a), E. coli (PDB: 6GV9) (b), N. gonorrhoeae (PDB: 5VXX) (c), T. thermophilus (PDB: 6XXD) (d), G. sulfurreducens (PDB: 6VK9) (e), M. xanthus (PDB: 8TJ2) (f), P. arsenaticum (PDB: 6W8U) (g). (TIF) [file ppat.1012773.s010.tif]

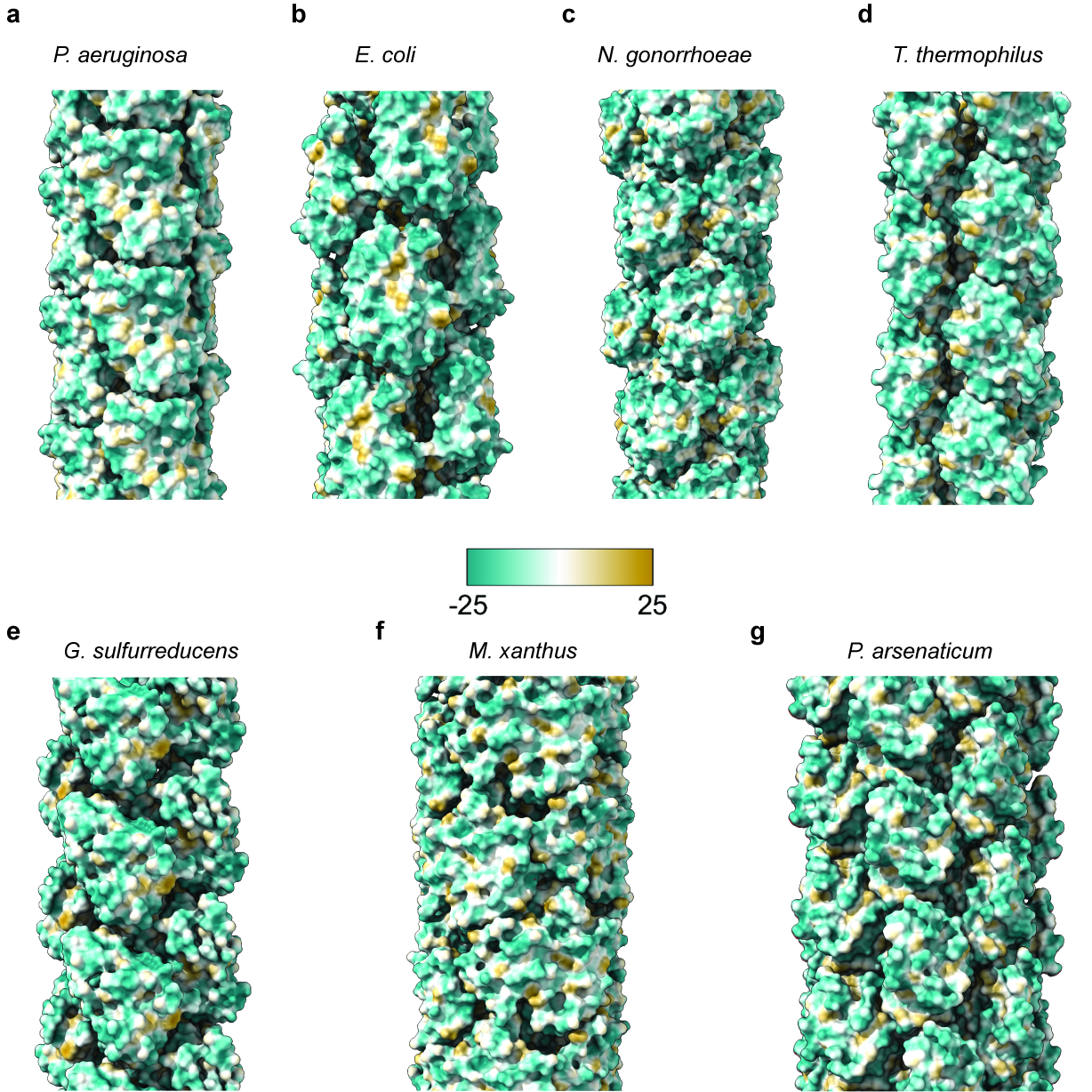

Supplement: S11 Fig — a-g Models of T4P structures from different organisms coloured by molecular lipophilicity potential using the mlp function in ChimeraX [86]: P. aeruginosa PAO1 (this study, PDB: 9EWX) (a), E. coli (PDB: 6GV9) (b), N. gonorrhoeae (PDB: 5VXX) (c), T. thermophilus (PDB: 6XXD) (d), G. sulfurreducens (PDB: 6VK9) (e), M. xanthus (PDB: 8TJ2) (f), P. arsenaticum (PDB: 6W8U) (g). Negative potential corresponds to hydrophilic regions, positive potential to hydrophobic regions. (TIF) [file ppat.1012773.s011.tif]
