# Supplementary material for: Structure of the Pseudomonas aeruginosa PAO1 Type IV pilus
Source: PLoS Pathog. 2024 Dec 12;20(12):e1012773. doi: 10.1371/journal.ppat.1012773 (PMC11670995; doi:10.1371/journal.ppat.1012773)
Supplement: S1 Table — (DOCX) [file ppat.1012773.s012.docx]

**S1 Table: Cryo-EM data acquisition and processing statistics**

| **Data collection and processing** | PilA ΔpilT [EMDB 50025, PDB 9EWX] |
| --- | --- |
| Microscope | Krios Titan G2 |
| Magnification | 96,000 |
| Voltage (kV) | 300 |
| Electron exposure (e^–^/Å^2^) | 40 |
| Defocus range (μm) | -1 to -2.5 |
| Pixel size (Å) | 0.824 |
| Symmetry imposed | Helical, final: 87.39° 10.17 Å |
| Initial particle images (no.) | 114,537 |
| Final particle images (no.) | 76,998 |
| Map resolution (Å)  FSC threshold | 3.17  0.143 |
| Map resolution range (Å) | 3.0-3.7 |
|  |  |
| **Refinement** |  |
| Initial model used | AlphaFold2 |
| Model resolution (Å)  FSC threshold | 2.6/2.8/3.2  0/0.143/0.5 |
| Model resolution range (Å) | n/a |
| Map sharpening *B* factor (Å^2^) | -42.7 |
| Model composition  Non-hydrogen atoms  Protein residues  Ligands | 23,943  3,289  0 |
| *B* factors (Å^2^)  Protein  Ligand | 28.74  n/a |
| R.m.s. deviations  Bond lengths (Å)  Bond angles (°) | 0.003  0.554 |
| Validation  MolProbity score  Clashscore  Poor rotamers (%) | 1.22  2.8  0 |
| Ramachandran plot  Favored (%)  Allowed (%)  Disallowed (%) | 97.16  2.84  0 |
