# Supplementary material for: Structure of the Pseudomonas aeruginosa PAO1 Type IV pilus
Source: PLoS Pathog. 2024 Dec 12;20(12):e1012773. doi: 10.1371/journal.ppat.1012773 (PMC11670995; doi:10.1371/journal.ppat.1012773)
Supplement: S2 Table — (DOCX) [file ppat.1012773.s013.docx]

**S2 Table: T4P from various organisms**

|  | *Pseudomonas*  *aeruginosa* | *Neisseria gonorrhoeae* | *Escherichia*  *coli* | *Geobacter sulfurreducens* | *Thermus thermophilus* | *Myxococcus xanthus* | *Pyrobaculum arsenaticum* | *Monoderm bacterium* |
| --- | --- | --- | --- | --- | --- | --- | --- | --- |
| Pilin sequence length | 143 | 156 | 140 | 104 | 125 | 208 | 140 | 137 |
| Pilus diameter | 51Å | 62Å | 60Å | 65Å | 70Å | 70Å | 80Å | 70Å |
| Helical  rise | 10.17Å | 10.1Å | 11.2Å | 10.4Å | 9.33Å | 10.0Å | 5.3Å | 11Å |
| Helical twist | 87.39° | 100.8° | 96° | 89.1° | 92.5° | 100.7° | 101.7° | 93° |
| Helical pitch | 41Å | 36Å | 42Å | 42Å | 36Å | 36Å | 19Å | 43Å |
| PDB ID | 9EWX | 5VXX | 6GV9 | 6VK9 | 6XXD | 8TJ2 | 6W8U | 8PFB |
