# Supplementary material for: Structure of the Pseudomonas aeruginosa PAO1 Type IV pilus
Source: PLoS Pathog. 2024 Dec 12;20(12):e1012773. doi: 10.1371/journal.ppat.1012773 (PMC11670995; doi:10.1371/journal.ppat.1012773)
Supplement: S3 Table — The values were calculated using two different methods: TM-align [91] and jFATCAT [92]. (DOCX) [file ppat.1012773.s014.docx]

**S3 Table: RMSD values calculated between the *P. aeruginosa* PAO1 structure (9EWX) and other T4P species.** The values were calculated using two different methods: TM-align^5^ and jFATCAT^6^.

| **PDB** | **RMSD (Å)** | **Aligned residues** | **Identity** | **Domain** | **Method** |
| --- | --- | --- | --- | --- | --- |
| 5VXX | 4.07 | 99 | 16% | Full | TM-align |
| 6GV9 | 3.35 | 110 | 18% | Full | TM-align |
| 6VK9 (chain H) | 3.33 | 54 | 5% | Globular | TM-align |
| 6VK9 (chain A) | 1.81 | 58 | 47% | Melted helix | TM-align |
| 6W8U | 4.28 | 67 | 12% | Full | TM-align |
| 6XXD | 2.61 | 104 | 6% | Full | TM-align |
| 8TJ2 | 3.52 | 96 | 21% | Full | TM-align |
|  |  |  |  |  |  |
| 5VXX | 3.02 | 108 | 14% | Full | jFATCAT |
| 6GV9 | 3.05 | 110 | 7% | Full | jFATCAT |
| 6VK9 (chain H) | 3.1 | 52 | 7% | Globular | jFATCAT |
| 6VK9 (chain A) | 1.85 | 61 | 46% | Melted helix | jFATCAT |
| 6W8U | 5.31 | 66 | 7% | Full | jFATCAT |
| 6XXD | 3.08 | 111 | 11% | Full | jFATCAT |
| 8TJ2 | 4.07 | 104 | 25% | Full | jFATCAT |
