# Supplementary material for: Structure of the Pseudomonas aeruginosa PAO1 Type IV pilus
Source: PLoS Pathog. 2024 Dec 12;20(12):e1012773. doi: 10.1371/journal.ppat.1012773 (PMC11670995; doi:10.1371/journal.ppat.1012773)
Supplement: S1 File — (PDF) [file ppat.1012773.s016.pdf]

```
>Input_seq_ATOM_A
FTLIELMIVVAIIGILAAIAIPQYQNYVARSEGASALATINPLKTTVEESLSRGIAGSKI
KIG---T-AA-S----TAT------ETYV-------GVE-PDA-----------------
--NKLGVI-------AVAIE-------DSGAG--------------DITFTFQT--GTS-
---SPK-N--ATK----VITLNRT--------------------AD-------GVWACK-
-----STQ-DP----MFTPKGCDN--
>ur|P17837|7_148|8.3e-70|Pseudomonadaceae
FTLIELMIVVAIIGILAAIAIPQYQNYVARSEGASALATINPLKTTVEESLSRGIAGSKI
LIG---T-TA-S----TAD------TTYV-------GID-EKA-----------------
--NKLGTV-------AVTIK-------DTGDG--------------TVKFTFAT--GQS-
---SPK-N--AGK----EITLNRT--------------------AE-------GVWTCT-
-----STQ-EE----MFIPKGCN---
>ur|UPI002738A414|7_140|5.9e-30|Photobacterium_leiognathi
FTLIELMIVVAVIGVLSAIAIPQYQKYVAKAEGAAALSTLTGLKTNVEAFTVEN------
--G---T-FP-G----ADD------ETAI-------GIP-------------A-------
--AELGEI-------SLPAA-----APTTAEG--------------AITFTFSD--GKV-
---SPL-N--TGT----SIALNRD--------------------TD-------GKWTCT-
----GTTD-DE----SILPKGC----
>ur|UPI0021C02504|7_140|1.7e-26|Acinetobacter_variabilis
FTLIELMIVVAIIGILAAIAIPQYQNYVGRSNFASGLSSLTALKTNIESQIMET------
--G---A-FP-A----NTD------LAADKL-----GMV-AGA-----------------
--NPNGTI-------ALAAV-----GTTGGAG--------------TSTFTFTK--GNPG
-------I--NGK----KIRLSRD--------------------AN-------GTWTCT-
-----SDA-DE----KYV-RGC----
>ur|UPI002738007C|10_139|2.2e-25|Shewanella_sp._NIFS-20-20
FTLIELMIVVAIIGILAAIALPAYQDYTVKSQAASALAEISGGKIGFEQAINQG------
--D---T-PS-T-G--TAD------EGYI-------GIV-AAT-----------------
--NYCA-V-------VLAT---------TAPY--------------KITCTAKG--GNA-
----DK-F--NGK----KIELTRT--------------------SD-------GVWACT-
-----SDL-DA----KFKPGKC----
>ur|UPI0024106262|7_155|9.8e-25|Dyella_jiangningensis
FTLIELMIVVAIIAILAAIAIPQYQTYVVKSQVTRAIAEAGDMKTTVEDCVNNG------
--H---L-TV-A-------------GGAGN-----CDPN-VTG---------S-------
--DILTGK------QQGSIP------DVANTGVPQLTINAD--GSGTIIATFGN--HA--
---SAK-LTSAAQ----TVEWDRT--------------------TD-------GTWTCK-
----ATGV-DV----KYLPASC----
>ur|UPI001EDDE431|7_145|6e-24|Photobacterium_damselae
FTLIELMIVVAVIGVLSAIAIPQYQKYVAKAEAASALATLTGLKTNAEAYTIEN------
--G---T-FP-D----GES------VNGI------LGAP-------------S-------
--TALGTI-------TFTADV--SDSKAQPTG--------------KITFAFAK--ANV-
---SPL-N--SGN----TLSLERK--------------------SD-------GGWTCS-
----SSSK-DT----GIIPKGCT---
>ur|A0A1E2YH21|7_161|2.2e-23|Moraxellaceae
FTLIELMIVVAIIGILAAIAIPQYQNYVGRSNVASAVQTLTSNKTGLENYVMEN------
--G---F-FP-D----GKK------PSQAEV-KDAQGNV-TTPYLP-DQRMKE-GLG-IV
Q-PSFGAI-------TLEQK-----STTAGTG--------------NIVLTFNT--GNPG
-------I--KGN----KVQLHRA--------------------ED-------GTWTCE-
```

-----TTI-DG----KYAAKSCS---
>ur|A0A1I4QPX6|8_146|6.5e-23|Marinobacter_zhejiangensis
FTLIELMIVVAIIGILAAVAIPQYQNYVSKSQVTRVMQETGALKTVIETCLLDG------
--L---T-AA-G----------------------ECELG-WTN---------S-------
--NLIGAT-------TVAG---------LQTGLTVI-LAEDNTEESSIEAQFGG--NA--
---SAA-V--AGE----TLTWTRD--------------------TD-------GSWACS-
-----TTV-ET----AFRPTGC----
>ur|A0A427CED5|8_136|1.6e-22|Stenotrophomonas_sp._278
FTLIELMIVVAIIAILAAIALPLYQDYMAKAQLGAALSEIRPGKTTMESVAQDS------
--T---D-AS-L-----VT------PDYI-------GVR--VS----------------
--ERCTSV-------AASLD-------ASGVG--------------TISCTVKG--GAA-
-------V--NGK----SLTLRRA--------------------AN-------GVWTCD-
----GSEF-AA----RYRPSGC----
>ur|UPI00143A8648|7_138|3.3e-22|Acinetobacter
FTLIELMIVVAIIGILAAIAIPAYQDYIAKSQATSGLAEISPAKTQYEVLVNEG------
--K---P-SS-D-----FT------VANV-------GLK-DS------------------
--DRCT-V-------AVTAP-----TSGAATN--------------AITCTLKG--SPK-
-------V--ENA----VINLGRA--------------------AD-------GTWACTL
---TGTTL-DA----KYKPGSC----
>ur|A0A840UE77|19_163|5.8e-22|Marinobacter_oulmenensis
FTLIELMIVVAIIGILAAIAIPQYQDYIARSQAAEAVNLLGGAKTPIEEYILVN------
--G---A-FP-D----GTT--SGQQLSDL-------GVR-DSG-----------------
--TYVDTL-------AIANP-------SNEDG--------------DIVATFKT--SDV-
---ASA-L--SGN----TIRLKRTVDA--------------T-TGE-------STWQCIT
---TDSDM-EQ----KYLPSGC----
>ur|UPI00135B5437|8_136|1.2e-21|Alcanivorax_sp._S71-1-4
FTLIELMIVVAIIGILAAIAIPQYQDYISRSQVQRVVGEASSVRTAIEEILMRG------
--G---N-PT-T-T--STD------EGPTD---FFVGFD-AAR---------S-------
--NLMSAF-------TVA----------NAAG------------VVDVEATLGG--NA--
---NSA-V--DGA----VITWSRD--------------------AE-------GVWTCV-
----TTAS------------------
>ur|UPI0028AF9543|9_151|2.3e-21|Pseudomonadaceae
FTLIELMIVVAIIGILAAIAIPQYQNYVGKSQVSRVMSETGAIKTAVESCMMDG------
--T---A-EA-D-----------------------CNIG-WAL---------S-------
--NLIGEG-------GVDVG--------EQEGLEVT-YDLDE-GSASIVAGFGT--NA--
---SSA-I--RGE----NLTWTRADNDA---------------DNA-------GSWSCT-
-----TSV-EN----KFRPSGC----
>ur|A0A009SB42|7_160|4.2e-21|Acinetobacter_calcoaceticus/baumannii_complex
FTLIELMIVVAIIGILAAIAIPQYQNYVGRSNVAAAVQTLTSNKTGLENYVMEN------
--G---F-FP-D----GKT------AEQAEV-K-TNGVV-TTPFIP-DQRLKE-GIG-IV
Q-PSFGTI-------ELQQK-----STTAGTG--------------NIVITFNT--GNPG
-------I--KGN----KVQLHRA--------------------ED-------GTWTCE-
-----TTI-DA----KYAAKSCS---
>ur|UPI00225902FB|8_142|7.5e-21|Dyella_silvatica
FTLIELMIVVAIIAILAAIAIPQYQTYVIKSQFTRIMGEASDQRVTVEDCLNNG------
--V---V-TI-G----ATG----------------CVLT-ATS---------S-------
--DLLST--------QVAIG------TLASAG-------------STITATFGG--HA--

---NPK-L--ATH----ILTWARN--------------------SS-------GAWSCK-
-----SDL-VATDKVKYAPVSC----
>ur|A0A7W3FLT2|7_136|1.4e-20|Stenotrophomonas_tumulicola
FTLIELMIVVAIIAILAAIALPQYQGYVSKAQLGAALADIRPGKTTMESVAQDS------
--R---D-AS-I-----VD------ANYI-------GLS--VT----------------
--TRCPII-------DAELA-------ADGVG--------------TITCTMEG--NSA-
-------V--DGR----DLVLRRT--------------------ID-------GTWTCD-
----ASAF-DA----EIRPSGCT---
>ur|A0A432LQI6|8_152|2.4e-20|Dyella_dinghuensis
FTLIELMIVVAIIAILAAIAIPQYQAYVIRSQVTRVMGEAGDQKVVVEDCLNNG------
--T---T-GT-I-T--PAG---GGAGGNTS----ACVET-ATS---------S-------
--ALMTGT-------EVPAI------VINTSG------------TASITSTFGG--SA--
---NTV-I--SGN----NLIWTRS--------------------AS-------GGWTCS-
-----TTV-SA----KYAPTSCPN--
>ur|A0A2I0GZ51|8_162|4.1e-20|Psychrobacter_sp._4Bb
FTLIELMIVVAIIGILAAIAIPQYQNYVGRSNVAAAVATLSSNKTGLEDYVLSN------
--G---A-FP-D----GKT------VAVQGK-PAGPGGT-PAAVLPVAGQTPA-DLG-IV
N-STIGET-------KIANV-----AATPGAG--------------KVQLKFAN--GNPG
-------I--KDK----IVQLERD--------------------AN-------GTWTCL-
-----TDV-EE----KFAGKAC----
>ur|UPI0027B3A80B|8_156|8.4e-20|Marinimicrobium_sp._ABcell2
FTLIELMIVVAIIGILAAVAIPQYQNYIASSQVSRVMSETGALRTAVENCLLQG------
--R---T-EV-T----TGD------PTATQ-----CNVG-WTF---------S-------
--NLLAGE-------AVQV---------QGDGLSIE--IED--NTATILATFGT--NA--
---AAA-LT-AGEGGAGQLQWFRN--------------------AS-------GVWVCS-
-----TNV-DM----RYRPGGCS---
>ur|A0A1I3PHW7|13_145|1.4e-19|Marinobacter
FTLIELMIVVAIIGILAAVAIPQYQDYTARSQVTRVVGEVNALKTNIEDALQRG------
--A---T-IV-D-G-YTDD------IANNQ---IALAYD-STK---------S-------
--NLLDGT------QGTNGV------TITDGGT----------ATPTIEAFLGG--QA--
---SAA-V--SGA----VVELSRD--------------------AQ-------GRWTCI-

>ur|A0A9E0U967|8_139|2.3e-19|Pseudomonadota_bacterium
FTLIELMIVVAIIGILAAVALPSYQNYTVQSQVKSALKEITPGRDQAEILLNKG------
--L---T-LS-T-N--PAD------TGFI-------GVQ-AST-----------------
--SYCN-V-------TIIGP-------GTTTP--------------TIRCTGSG--GNL-
----AK-F--VGK----TIDWVRD--------------------VN-------GKWTCV-
-----SNL-EP----KFKPRAC----
>ur|UPI002449C303|8_160|3.6e-19|Acinetobacter_johnsonii
FTLIELMIVVAIIGILAAIAIPAYQNYTAKSQASVALADITGAKVNIEAKLSEG------
--I---N-AA-T-AAGLTS------GNQV-------GVK-NVT-----------------
--STCNGV-------GVAMS-------AEGAT--------------AVTCKVKG--SAK-
-------V--NDL----YISWVRSSDKNAIVN------TTTP-ASS--VDEQTGSWECL-
-----TTI-PA----TLAPKNCVN--
>ur|UPI0010FED564|7_131|5.4e-19|Photobacterium_damselae
FTLIELMIVVAVIGVLSAIAIPMYKDYVKKSEVAAGIATLKPLMTEAELVYQQN------
--G---S-LS-A----GADT-----LTTL-------GIS-AAA-----------------

--NPLGTI-------SIKTGN-------------------------VIEFAFDT--NKSA
-------V--SG-----ALSLTRD--------------------ET-------KGWSCS-
-----NTT-------SISVKGC----
>ur|A0A7Y6NJV7|9_146|8e-19|Schlegelella_koreensis
FTLIELMIVVAIIGILAAVALPAYQDYIAKSQVTAGVAEINPGKTKIEDIYGGS------
--A---A-AS-G-----TD------VTQL-------GLK-ADT-----------------
--DRCA-I-------TMTGF-----AAVGDAG--------------TIVCTLKG--NGQ-
-------V--NGK----TVTWSRT--------------------ADAPATGATGIWSCA-
-----SNA-AR----KLTPKECP---
>ur|A0A084IQ36|7_144|1.1e-18|Salinisphaera_hydrothermalis__strain_C41B8
FTLIELMIVVAIIGILAAIAIPQYQNYVARSKVTEGINLASSLKTTVAENAMNG------
--S---D------------------DLSK-------GAE-TGQ---------T-------
T-DIVSGL-------SVD----------SSTG--------------AITVSYTD--KIPG
-------T--GAK----ELILTPKSGGKA-----LSA-GTPP-TNS-------ITWTCS-
----TNGL-DS----AYVPANC----
>ur|UPI0018E62E44|7_134|1.7e-18|Pseudomonas
FTLIELLIVVAIIGILATFALPQYSRYQARAKATAGLAEVSALRVAFEDVMNQG------
--T---N-PT---------------LANI-------GGT-SPT-----------------
--SNCT-M-------TVSGT------ASAGTG--------------SIACALLN--APAP
-------V--LGR----TITLSRS--------------------AE-------GVWTCA-
-----STA-AQ----EFLPAGCT---
>ur|UPI00273B64D9|13_147|2.5e-18|Marinobacter_salarius
FTLIELMIVVAIIGILAAVAIPQYQDYTARTQVSRVVGEVNALKSSAESIFNSG------
--G---A-VA-N----ADN------LSATP--KREIGIG-WTG---------S-------
--NLVDPT------HGTNGL------NITNGGQ----------ADMSWDVVLGT--DA--
---SGA-V--AGV----TITVSRS--------------------TS-------GAWSCAI
T-G-----------------------
>ur|UPI00071D5531|8_158|3.4e-18|Vitreoscilla_massiliensis
FTLIELMIVIAIIGILAAIALPMYQDYISKSQITRVVGELAAAKTGIDAALFDG------
--K---T-PV-----LDEG------TTATT--EVGMGLQ-TTG-----GTVRS-------
--NLINTL-------ATTFA------AGTGAG--------------SVTATLGR--NA--
---NAD-I--HTA----TIAQNRD--------------------AQ-------GVWTCHI
QAGTATGW-KD----KFIPNGCEN--
>ur|UPI00124EC2D5|7_125|4.8e-18|Acinetobacter
FTLIELMIVVAIIGILAAIAIPAYQNYIARSQASEAFTLADGLKTTINTNLQAG------
--T---C-FA-G------------------------GANAATA---------ADKVG---
--GKYGDA-------EIGGT--------APSC--------------TITYKFKS--SGV-
---SNK-L--TGK----TIGMNVS--------------------ET-------GILTKN-

>ur|A0A2N8REJ2|9_139|6.9e-18|Stutzerimonas_stutzeri
FTLIELMIVVAIIGILAAIAIPAYQDYVAKAKANAAYADIAGGKTGYEMAAVEG------
--S---A-AT-P----AAY------LEKA-------GLP-TAT-----------------
--GNCSTI-------AAAVP-------GANTA--------------VLTCTIAT--SGR-
-------LG-ATA----TIALHRS--------------------TK-------GLYSCV-
-----TAI-DE----KYRPAGC----
>ur|A0A7W7KSV3|7_134|1e-17|Xanthomonas
FTLIELMIVIAIIAILAAIALPMYQDYVAKSQVTAGLAEINPGKTQYEVALNEG------

--K---T-SI-T-G-----------IGDL-------GLR-SPT----------------
--DRCV-I-------TDITT-------LSATG--------------TIVCTLKG--NTQ-
-------V--KDK----TVTLTRR--------------------TD-------GTWTCA-
-----TSA-AA----KHAPAGCP---
>ur|A0A562PWM6|12_107|1.4e-17|Pseudoduganella_flava
FTLIELMIVVAIIGILAAVAIPAYQNYVKRAAYAEVVSQISPYKLAVEEAFQNG------
--V---A-LA-D-M----D------AATNAG--AATNIP-ATP---------T-TST---
T-GVFNSL-------AV------------ADG--------------VITA----------

>ur|A0A8I2BVU4|7_140|2.1e-17|Xanthomonas
FTLIELMIVIAIIAILAAIALPMYQDYVAKSQVAAGLAEITPGKVNAETRIAEG------
--K---A-AT-T-T-----------AADV-------GLA--TS----------------
--PRCTAL-------VVSVD-------PSGTS--------------SIACTLAG--NAQ-
-------V--ATK----TVTWSRT--------------------ADNATTGASGVWSCA-
-----TTV-AA----KLKPATC----
>ur|A0A542M1E6|14_148|2.7e-17|Herbaspirillum_sp._SJZ107
FTLIELMIVVAIIGILAAVAIPAYQDYIAKSKATAALADITGGKTGYEMAAVEG------
--T---A-TM-T----ADD-----YKAKT-------GLS-AST----------------
--GNCSSI-------TVATP----VTTGAAAA--------------VITCAINN--PGR-
-------LG-AAS----TIALYRS--------------------AE-------GKYSCV-
-----TAI-DQ----KYRPTGC----
>ur|A0A3B7M152|8_139|3.7e-17|Acinetobacter
FTLIELMIVIAIIGILAAVAIPAYQDYIAKSRASVALHEISAGKSSYELYVNHQ------
--Y---S-TILT-------------PADI-------NLS-SST----------------
--PTCT-I-------SATNP----DNTGVALK--------------AIRCHLKN--TTG-
-------FS-ANA----EIYINRS--------------------AD-------GIYSCG-
----TTGF-PE----KYKPNGC----
>ur|A0A3A8EV59|8_115|5.1e-17|Acinetobacter_rongchengensis
FTLIELMIVVAIIGILAAFAIPAYQDYIARGQAAEGFSLADGLKTSIADDLQNG------
--T---C-GS-S----SAH---------------------------------------
G-KYAASV-------KVTDV-------APNNG------------ECVITIVYAT--GI--
---ADQ-V--QGK----RVVAKTNKNG--------------------------------

>ur|A0A098PXD3|12_148|7.3e-17|Xanthomonas_axonopodis
FTLIELMIVIAIIAVLASIAIPQYQIYVGKSQVAASLAEVNPGKTRYEILVNDG------
--D---G-SI------LTS------AAVI-------GLTVTAT----------------
--SRCSGI-------SVLAP----DANGAQTH--------------AIDCTVRG--NPR-
-------V--NGA----KVTWARD--------------------AS-------GIWTCS-
-----TDL-ASTDKDRFAPASC----
>ur|UPI0007F9224F|7_130|9.7e-17|Photobacterium_phosphoreum
FTLIELMIVVAIIGVLSAFAIPAYKSYVAKSEASTGVATLRALLTNYDMYLQEN------
--G---E-AP-T------D------LTQI-------GSS-SGA---------S-------
--SSLGEI-------ALTET-------------------------SGATFTFQG--SGA-
-------L--SGA----VVTYTR---------------------SN-------AGWKCT-
-----STI-AD----DYKPKGC----
>ur|A0A2N8T3S5|9_136|1.4e-16|Pseudomonadaceae

FTLIELMIVVAIIGILAAIAIPQYQNYIGRSQFSEAHALLSGAKVAVQEKIDQG------
--E---A-II-G-----TD--------------LELQLA--------------------
--GTYGDI-------TDPAD--------AAAGA----------TTYSLVYIFDN------
--ANPS-L--NGS----RVTYAYD-------------------QAT-------GKWTCT-
-----TDA-DA----KFVTK------
>ur|A0A5Q4Z405|10_123|1.7e-16|Aliivibrio_wodanis
FTLIELMIVVAIIGVLSAIAIPAYKDYVSKSEAASGLATIKALQTPAELFFQEK------
--G---T-GT-A----PA-------LDAL-------GTT-TGA-----------------
--NALGLI-------TATT------------S--------------TIVFTFGA--ES--
-----A-L--SGK----ALTLTRD--------------------AS-------TGWTCA-
-----AA-------------------
>ur|A0A3A8G6I8|7_142|2.2e-16|Acinetobacter
FTLIEMMIVVAIVGVLAAISIPLYQGYVAKSQITTALAELNGAKTQYELILSGA------
--T---A-SS------TTD------FTVVN-----MFFS-GSQ-----------------
--SNVCIY-------DVNPP-----ASGVANK--------------ALVCQLNH------
--VATP-I--AGE----FVYLNRD--------------------LA-------GAWTCS-
-----TSLGIAT---KFKPSGC----
>ur|A0A5P9C5A9|9_137|3.1e-16|unclassified_Pseudomonas
FTLIELMIVVAIIGILAAIALPAYQDYVAKSQVTAGLADIRGGVTAFEEIIQSG------
--A---S-RP-------AN------AADI-------GLQ-AST-----------------
--ARCT-I-------SITAT--------AGSE--------------EISCALKG--NPK-
-------V--ATK----TVDLVRN--------------------SS-------GAWNCVV
----DTTI-DS----KYRPVGCN---
>ur|UPI0002A57025|12_124|4.1e-16|Vibrionaceae
FTLIELMIVVAIIGVLSAIAVPAYQNYVAKSEAATALGSLRALVTPAELKLQQD------
--G---E-LS-G-V-----------VADL-------GGS--AS-----------------
--HALGAI-------TTSGA-------NISAA--------------TLTFTFNT--GS--
-------L--SGD----AITLTK---------------------TS-------SGWTCT-

>ur|A0A562D7B2|7_138|5.6e-16|Pseudoxanthomonas_taiwanensis_J19
FTLIELMIVVAIIAILAAIALPAYQNYVAKSQVTAGLADIRGGVTAYEELIQSG------
--T---K-SG-K-----PD------LDDL-------GLA-SST-----------------
--ARCA-I-------TADGN-----IEDKKDQ--------------SIACELKG--NPK-
-------V--VGE----TIKLVRT--------------------EE-------GAWNCVI
----DGDL-DE----KFWPAGC----
>ur|A0A7T0RHY0|17_142|7.6e-16|unclassified_Halomonas
FTLIELMIVVAIIGVLASIAVPQYQNYTARAQASEGLSVTAGMRADIAEQYSLQ------
--G---G-MP-S----TDD------IDDL-----------VETDDE-----PA-------
G-RYVQNA-------TYAFA--------EDTG--------------TITVTFDT--DSA-
-------L--GNR----TMLLETD-------------------NPQ-------DGWVCKA
----GTTD------------------
>ur|UPI001ADAE79F|7_137|1e-15|Xanthomonas_sp._D-109
FSLVELMIVVAIVAILAAIALPLYQGYVARAQVASALDEITIGKVGYETLISQG------
--A---G-DS-S-----YT------NDRL-------GVH-TST-----------------
--VRCSQI-------SVYAP-----SGGSAMP--------------AISCSMVG--SSG-
-------V--DGE----VIRWDLA--------------------ND-------DVWSCS-
-----TTV-DA----RYAPVGC----

>ur|A0A375J2J3|86_218|1.3e-15|Cupriavidus_taiwanensis
FTLIELMIVVAIVGILAAIAIPQYQDYVTRSRWSENITSVAALKMAVAQCLQEN------
--G---GVLA-S-C----D------TVARLT--ATVGYP-GIQ---------N-------
--PPNGAV-------ALT----------PATA--------------AIVITGAA------
------AL--PAC----TVTMAPVVGT-----------------NA-------VTWNMA-
----TGGG-----------ATCTRAQ
>ur|UPI0003615C2F|11_122|1.6e-15|Methylotenera
FTLVEIMIVVAIIGILASVAIPSYQDYVKKGKAAEATATLADLRIKMEQCFQDN------
--R---S-YA--------------------------GCA-AFC---------A-------
--PTSGAV-----NFSYACA--------ATPD--------------ALTYNIVA--TGVS
---GKG-M--TGF----SYSVNQSNAK----------------T----------------

>ur|UPI00257B592B|7_150|2e-15|Psychrobacter_sp._UBA6291
FTLIELMIVVAIIGILAAIAIPQYQTYTAKSQVTRAVSEAAGLKTALDTCILDG------
--K---A-TT-A-----------------------CDFG-ATG---------S-------
--NILTAQ-------TTGGLNGTNGAVTGKAGVPAASIGANATTNSTITGTLGF--SA--
---APA-V--AGD----TVVWTRD-------------------AAT-------GTWTCA-
-----SDV-LK----KY---------
>ur|C6M3S4|8_158|2.6e-15|Neisseria
FTLIELMIVVAIIGILAAIALPMYGDYTARAQATEGYELLGGMKTPLVEAVAAT------
--S---NDVA---C---------NGTAAT-------GTA-AATDPA---WFKSAVLS---
G-KYVAGV-------KAEHA----GQDDAATC--------------TLTATFKA--ASAG
--VNDK-V--AGK----TISMKLT-------------------PAT-------GAWECD-
-----TTL-GD----NVAPAAC----
>ur|A0A2G4WNG1|7_127|3.3e-15|Photobacterium_leiognathi
FTLIELMIVVAVIGVLSAIAIPKYQEYVKKAALGTALASLSAYKTNIEDTIATT------
--G---D-FT-S----AAD--------------------------------KT-------
--FGIGKI-------TFTPG-------TSSAG--------------SIQATIEE--GSA-
----------INQ----TVTLSRD--------------------AA-------GIWSCA-
-----HSL-SA----SINITGC----
>ur|A0A0A7JRD7|8_133|3.9e-15|Pseudomonadaceae
-TLIELMIVVAIIGILATIAIPMYTNHQARGKAAAGLLEISALKTPMDLRLNEG------
--K---D-VT-D-------------AASL-------GGQ-AST-----------------
--AHCA-I-------LASGT------AAAGTG--------------SIVCTLLD--APAN
-------V--LGK----RLTLTRS--------------------AT-------G-WACS-
-----TDL-ES----DLAPSGC----
>ur|R4YQ02|8_103|4.6e-15|Oleispira_antarctica_RB-8
FTLIELMIVVAIIGILASVSIPMYRDYIVRTKVATAMATVSNIKTAIALTNNEG------
--I---A-VP-A-I--AAG------AAKAE--WQKIGMR-NEP-----------------
--KFSSEV-------KSAAI--------AAGG--------------EIT-----------

>ur|A0A1A9RMA8|8_140|5.8e-15|Eikenella_corrodens
FTLIELMIVIAIIGILAAIALPAYQDYVARSQLGEAFTLASGQKGAVAESYSDK------
--G---V-LP-A------D------NTAA-------GIA-SAT---------S--IK---
G-KYVAKV-------EV------------ANG--------------VITATMNA--TGV-
---AAG-V--QGK----IITLTPTVNK-----------------GS-------FSWACS-

----S-DA-DA----KYRPSSC----
>ur|B2UGE5|16_147|6.8e-15|Ralstonia
FTLIELMIVVAIVGILAALAIPAYQDYTIRSRVTEGLIIAVQAKALVVENAANA------
--Q---S------------------SLSL-------GAA-SLS---------A-------
T-RNVASL-------DID----------GSSG--------------EISIGFTS--SVAG
-------A--GSN----TLILTPYSGTASSTYANLSA-GSAP-PAV-------VMWACSA
N------------------------
>ur|UPI000382BBCC|10_140|8.1e-15|Thioalkalivibrio_sp._ALJ16
FTLIELMIVVAIIGILAAIAIPAYQDYTARAQASEALSVTAGLRADIAERVALG------
--Q---V----D----NLD-----------------NIV-ETN---------T-DLA---
G-RYVATV-------DV-----------DEDG--------------LITVVFGT--AGV-
---ANP-I--SGA----TMLIGPVNTAGDEIIDV----DDTI-DRI-------EGWGCE-

>ur|A0A369UVX3|9_149|9.5e-15|Dyella_tabacisoli
FTLIELMIVVAIIAILAAIALPAYQDYVVKSRVSEAMVLADGLKAVVAENAATG------
--A---T-GA-G-------------AFSK-------GAT-LTT---------A-ADK---
S-PNVQTT-------SIS----------DTTG--------------AISVLTQA--RA--
----------GNG----TVVFTPTANNAALVA------GTPP-QNI-------IVWTCA-
----SADI-KQ----KYLPASC----
>ur|UPI0002FA76FD|11_121|1.2e-14|Vibrio_genomosp._F10
FTLIELMIVVAIIGVLSAIAVPAYKDYVTKSQASSALATIKSLVTSAELIIQEE------
--G---S-IS-G------------GVDLL-------GIS-SSS-----------------
--STLGTL-------SIVD----------SGK--------------SIQFEFSA--GA--
-------L--ASQ----TMKLAR---------------------SD-------SGWTCT-

>ur|A0A925S5C3|7_167|1.4e-14|Rhizobacter_sp.
FTLIELMIVVAIIGILAAIAIPQYQTYVAKSQVSRVMYEASSLKSVAETCVLEG------
--R---T-GA-----FFSNIVAGGAQGATD-----CNLQ-ATA---------S-------
--SLLFGA-------AVGPD---ASPAPPGTGYPIA-TMVG--GLVNVTATFGN--GA--
---AGV-LA-TGP---GILRWQRD--------------------TS-------GAWTCAV
----NAGV-DA----RYRAKGCD---
>ur|UPI00244A1D30|8_103|1.6e-14|Acinetobacter_johnsonii
FTLIELMIVVAIIGILAAIALPAYQNYTVRAKVSEVVLAASACRTTITETIQSAVG----
--S---T-LP-A-------------ANAW-------GCE-SAS---------A---T---
T-KYVASV-------AT-----------DNKG--------------VITVTAQN--IS--

>ur|UPI0011A13069|7_126|1.9e-14|Marinobacter_maritimus
FTLIELMIVVAVIGILAAIAIPSYQGYIIKTQTNRAVGELSAYRAPFEERTGSG------
--G---S-VT-N-----------------------NDIG-YTP---------S-------
--DLTNGV------QAVNIG----TLNPDGSG---------------QLQVTMGG--NA--
---HPN-L--TGL----ILRFDRD--------------------AS-------GRWECVV

>ur|UPI000415844F|8_133|2.3e-14|Pseudomonas
-TLIELMIVVAIIGILATIALPMYTNHQARSKAAAGLLEISALKTPMDLRLNDG------
--R---D-VT-D-------------VASL-------GGQ-PTT-----------------
--AHCA-I-------TASGK------AADASA--------------SIVCTLVD--APAN

-------V--LGK----GLTLTRS-------------------PS-------G-WACS-
-----TDI-EE----ELAPKGC----
>ur|A0A8E4F761|3_146|2.7e-14|Acinetobacter_terrae
--CIELMIVVAIIGILAAIAIPAYQDYIARAQASEAVNLAGGLKGAVSEVYSQD------
--G---S-CP-SNA--ATS------TTAN-------GIG-APS---------T--IT---
G-KYVASV-------TAAGT-------GTAAG--GC----------TITAKMKS--VGV-
---STG-I--QGK----SLILTLTNEG-----------------GS-------NSWACT-
----SADM-AQ----KYLPKSCQ---
>ur|O54650|8_152|3.1e-14|Dichelobacter_nodosus
FTLIELMIVVAIIGILAAFAIPAYNDYIARSQAAEGLTLADGLKIRIADHLENG------
--S---C-TE-D-A-----------AAAS-------GEK-GNE---------------DI
G-KYGKAVISGTYNEAATEP-------DAENGC-------------IVTITYGE--GTAK
DKVSKL-I--KGK----DLILSQL-------------------VN-------GSYTQS-
----GGTV-DP----KFVPN------
>ur|UPI0015D3DA80|3_134|3.6e-14|Acinetobacter_sp._YH12126
Y--IELMIVVAIIGILAAIAIPAYQDYIARSQMTEAMSLASGQKTNVTETYGQI------
--G---T-FT-G-I----S------SGSN-------GIP-AAT---------D--IT---
G-AYVAQV-------AV------------ADG--------------VITATMKG--TGV-
---SAG-I--VSK----TLTLTPADAT-----------------GS-------VTWTCS-
----S-NA-AQ----KYVPKAC----
>ur|A0A3A6NZF0|14_130|4.2e-14|Oxalobacter_sp.
FTLIELMIVVAIIGILAAIALPAYQDYTIRARVSEALVVAGAAKTTVSENISNN------
--G---G-IL-A------A------DNCL-------GVT-QIA---------A-AVA---
N-SNITSM-------TCA----------AATG--------------FITITTGA--NA--
----------GNV----TLDLRPQAPGAAGAVI--------------------------

>ur|UPI000379B462|12_155|4.9e-14|Methylosarcina_fibrata
FTLIELMIVVAIIGILAAVAIPAYKDYTTRAKMSEVLVMMEPAKLAVSETVSSM------
--G---S-LA-AFI----T------AGGTG---PVAGYN-FPG---------D-------
T-PYVDDI-------VV-----------ANDGT-------------VVTVTATSLVPG--
----------AQG----PVVLTAREVTGAA--------AGVG-SGQ-------LTWACT-
-----STI-DG----KYLPSNC----
>ur|A0A2U8GQT8|8_123|5.6e-14|Parazoarcus_communis
FTLIELMIVVAIIGILAAVAIPQYQDYVTRAKLSKVNVAVDPVKTAVAMFAQEN------
--A---G-VG-T-I-PANG------WTSL-------GLA-GAP--------TA-------
T-TEVSGI-------TVT----------AATG--------------AIVATLRS------
--IGTG-Y--DGS----TVTYTPTVGN--------------------------------

>ur|A0A6M8T0A4|8_123|6.7e-14|Deefgea
FTLIELMIVVAIIGILAAVAIPSYQDYTAKAKFTSAQAEIAAGKTGFDSQLNDG------
--V---A-IT-A-D--AAG------LALV-------GLK-GST-----------------
--SNCT-T-------TVTGS--------------------------TIVCTIVG--GPAT
-------V--AGK----KITLTRA--------------------ND-------GAWTCA-
-----SD-------------------
>ur|A0A931H8I0|9_139|7.6e-14|Caenimonas_aquaedulcis
FTLIELMIVVAIIGILAAVALPAYQDYTKRAKMSEVVLAASACRTTITEVYQSG------
--S---T-TP-A-------------ADGW-------GCE-SSS---------Q---T---

S-KYVKSI-------HT-----------DSNG--------------VVTVEATG--FND-
---TN--I--DDK----KIVLTPYVSDTATSAMP----ANRG-SAV-------FKWVCG-

>ur|UPI0005D87576|15_140|8.7e-14|Ralstonia_mannitolilytica
FTLIELMIVVAIVGILAAIAIPAYQDYTIRARVTEGLSLAAAAKVLVAENAANA------
--Q---S------------------DLSA-------GSA-PFV---------A-------
T-KNVKNL-------QIN----------GTDG--------------HITITYDT--NA--
----------GNG----DLTITPYYNNSGTLTN-LAT-SSVP-TAP-------VQWRCA-

>ur|UPI001C81E6DD|15_115|1e-13|Noviherbaspirillum_aridicola
FTLIELMIVVAIIGILAAVAIPQYQDYTVKAKIANALSAVDTLKTAVALCAQEA------
--G---GSVD-G-C----D------AGSN-------GVP-ATN---------A-FTP---
T-KEVAAL------NSI-----------TNG--------------VITIRLSA--TGM-
---G---

>ur|UPI000C818471|13_113|1.2e-13|Vibrio
FTLIELMIVVAVIGVLSAIAIPKYQEYVEKGALGSALATATALKTNYEDYLAVS------
--G---A-EP-T----SSS-----------------AIG-ATN-----------------
--FKLGSI-------DVTS-----------AG---------------IDVTITN--GG--
-------G--SGS----AVKLVRN--------------------G---------------

>ur|A0A952J123|8_108|1.3e-13|Granulosicoccus_sp.
FTLIELMIVIAIIGILAAVAVPQYQTYTARAKFSEVILATTPFKSAIEVCAQTN------
--G---A-LT-N-C---TT------AGAN-------GIP-PVP-------TAA-ALT---
A-GHVGSV-------AVALN--------GTDA--------------EITATAK-------

>ur|A4G8A4|14_131|1.5e-13|Herminiimonas_arsenicoxydans
FTLIELMIVVAIIGILAAVAIPQYQDYVTRAKLAKVNVAVESVKTAIALYAQEN------
--N---G-VA-D-I-TANG------WDSL-------GLA-GAP--------TA-------
T-EVVSGI-------DVT----------AATG--------------AIVATITG------
--VGSP-F--NGT----TVTYTPTVGNSA------------------------------

>ur|UPI001C44CBCC|8_132|1.7e-13|Acinetobacter_sp._CWB-G5
FTLIELMIVVAIIGILAAIAIPAYQNYTIRAKISDGLSLSSALKTAINESFQSK------
--G---P-SS-M------A------CSDAAT-CKAIGTNPLDS---------T-ALA---
GNANVTSV-------TS-----------VASG--------------IISIAYKP--AVL-
-----P-S--GSN----TLTLNPVAADGTTA----------------------------

>ur|A0A519I4S1|11_138|1.9e-13|Rubrivivax_sp.
FTLIELMIVVAIIGILAAVALPQYKDYTSKARIGNAISVVDPWKSAIALCAQEN------
--A---GALT-T-C----T------AGSG-------GVP-ADT---------T-KAP---
S-KEVSDV-------KT-----------GTNG--------------VVTFTFSE--AA--
---GGD-L--NTK----TVTFTPTVGE-----------------NS-------VTWAIS-
-----TTV---------------D--
>ur|UPI0015D1A12C|8_115|2.1e-13|unclassified_Acinetobacter
FTLIELMIVVAIIGILAAVAIPAYQDYTKRAKVSEGIALASAAKTAVVENAASA------

--A---V-Y------------------SL-------GYS-APT---------A-------
T-KDVTSV-------LID----------DENG--------------EITITYAA--PVQ-
----------AGG----TLILRPYTGTAA-----------VP------------------

>ur|UPI00145C99D4|8_138|2.4e-13|Aromatoleum_petrolei
FTLIELMIVVAIIGILAAVALPAYQDYTTRAKVSEVIVMSAPAKLAVSETASSL------
--G---S-LA-L-V----T------ASNS-------GYT-FPG---------A-------
T-KYVSNI-------AIA----------DTTG--------------VVTVTSTV--PG--
----------ATG----DLTMTPTVVG----------------GGQ-------LKWACA-
----STAI-NS----KFLPSEC----
>ur|A0A1J1E2W4|7_110|2.7e-13|Lysobacter_enzymogenes
FTLIELMIVIAILGILIAIALPAYQNYTIRTKNAECLNVAAGAKLAVAETAQDR------
--G---S-LA-A-I----T------ATNT-------GFQ-FQP-----------------
S-KYCNSV-------TVT----------ATTG--------------VVVAVSRD------
-------T--GGP----NVTFSLT-----------------------------------

>ur|UPI0008A5F514|8_141|3e-13|Neisseria_sp._HMSC072B12
FTLIELMIVIAIIGILAVIALPAYQDYTARAQVSEAISLMEGQKSAVVEYYADK------
--G---A-WP-A------N------NEQA-------GIA-TNT---------S--IQ---
G-KYVAQV-------DV-----------GANG--------------VITAKMKT--DDV-
---NNE-I--KGK----TVSLTPHATTAGTGNA-----AATA-NGS-------FTWTCT-
----S---------------------
>ur|UPI00293D4331|15_122|3.5e-13|Janthinobacterium_sp.
FTLIELMIVVAIIGILAAVAIPQYTDYTIKAKVGNALASAGPLKTAVALCIQEN------
--G---NNKA-G-C----T------TTGG-------TVT-SIP---------A-FTA---
T-KEVTSA-------TV------------TDG--------------EITMTLAS--GI--
---GSG-V--DGL----VIT---------------------------------------

>ur|A0A1I6ZN88|7_130|3.9e-13|Pseudomonas_marincola
FTLIELMIVVAIIGILAAVALPAYQDYTVRARVTEGLSLASSAKTTVSENAANG------
--N---A-F------------------AL-------GYT-APA---------A-------
T-DNVASV-------TIA----------AATG--------------EITISYTA--KA--
----------GNG----TLVLSPRDGGPAGAAL--AA-GTVPANGS-------ITWNC--

>ur|A0A8T7CS22|7_150|4.5e-13|Gammaproteobacteria_bacterium
FTLIELMIVVAIIGILAAIAIPAYQDYTQRAQIGEAFTVVSGAKTAIAEFAQTN------
--G---D-YP-T----GTD------ITAL-------SLD-IAS---------G--AA---
G-KYVDTV-------TVT----------ADTG--------------EISAVMKG--AGS-
--VGAK-I--AGT----EVQFCPPSQDLST-----------V-TGA-------FTWSCS-
----S-DA-EQ----KFLPKSCT---
>ur|A0A9X0N6Q7|8_112|5e-13|Neisseria_flavescens
FTLIELMIVIAILGILAVIALPAYQDYTIRAKVSEGLGLAAPAKLAVVETSAAL------
--G---G-LS-K-V----T------AENN-------GYK-FAT-----------------
T-KYVQSI-------AV-----------QDGG--------------VIKVVTKD--TGA-
-------T--TNP----AFTLTP------------------------------------

>ur|UPI0029536718|10_98|5.8e-13|Methylomarinovum_caldicuralii

FTLIELMIVVAIIGILAAIAIPAYQDYTKKAKAAELLNAAGPAKAAVSEFIVAN------
--N---S-LP-S------N------ATQA-------GFS-TVS-----------------
T-DYVSSV-------SWD----------SSNN--------------RIAVV---------

>ur|A0A3N7EMD5|8_122|6.6e-13|Paucibacter_sp._KBW04
FTLIELMIVVAIIGILAAVAIPQYKDYTAKTKAASAVSSMDGVKKAVALCAQEN------
--G---T-TT-G-C----S------SGTN-------GIP-AWS---------A-------
T-DVLTTV-------------------VATDG--------------TIVATFTP--AAK-
--VGAD-L--GDQ----TITFAPSLNASR------------------------------

>ur|A0A3E1DHQ6|8_104|7.3e-13|Pseudomonadota
FTLIELMIVVAIIGILAAVAIPAYQDYVVKAKLAKVASAVDPIKLAVATYMQEN------
--G---S-AA-G-L-TGND------WTTL-------GMS-AAP--------TA-------
T-TEVTGI-------TVT----------ATSG--------------AIVATLC-------

>ur|A0A4U6R2G6|8_130|8e-13|Marinobacter_panjinensis
FTLIELMIVVAIIGILAAVAIPAYQDYTARAQVSEGVSLAGGLKSTVSDIWSTK------
--G---A-LD-D-A----D------SNTN-------GLP-AAA---------D--VN---
G-SYVNDV-------SV------------TDG--------------LITVTMKS--SDV-
---SAG-I--QSA----TFLLSPITGS-----------------GS-------MIWTCK-
----T--------------------
>ur|A0A9X3UZJ7|9_119|8.7e-13|Comamonadaceae
FTLIELMIVVAIIGILAAVALPAYQDYTVRARVSEVILAASSCRTTVTDTVQNAP-----
--G---SDVS-S-------------ALNN-------GCS-ITA-----------------
T-KFVTSG-------ST-----------DSNG--------------VITVVGNA--TNL-
---KGD-VTTSAN----SIMLKPY-----------------------------------

>ur|A0A7V8JQH3|9_155|9.9e-13|Paracidovorax_wautersii
FTLIELMIAVALIGVLATVAIPQYQGHVARAQITRVVSETAALRPQAESCLLEG------
--K---T-NV-T----FSD----NEASAST-----CLIG-ATL---------S-------
--NLLGKT-------GQSEQ--------PPTGYPQI-VLAD--NETRITATFGN--TA--
---ATA-L--ANA----TVVWVRN--------------------AS-------GAWRCQ-
-----STV-AK----RYETSAC----
>ur|UPI0019611C71|8_116|1.1e-12|Spongiibacter_marinus
FTLIELMIVVAIIGILAAVALPAYQDYTIRAKVAEGLTLSSALKTAITETFQSQ------
--G---P-RD-M------S------CTDAAT-CGALGASVLDT---------T-ALA---
GNINVDSI-------TS-----------GETG--------------VITITYET--SVL-
-----P-A--T---

>ur|F2GCY9|12_122|1.2e-12|Alteromonas_mediterranea
FTLIELMIVVAIIGILAAIALPAYQTYTARATYSEVISASSAAKTAVEVCAQTG------
--V-----PA-D-C---TD------IAVNA------GWT------------NA-------
--STVNTI-------EIGGD--------AANGY-------------TITVTPNE--ESG-
-------IV-AAD----TLILTGTI----------------------------------

>ur|A0A127JSZ0|9_110|1.4e-12|Ramlibacter_tataouinensis
FTLIELMIVVAIIGILAAVALPAYQDYTVRAKNSEVILAASGCRTSITEVVQSA------
--S---T-LP-T-------------ANNW-------GCE-SAA---------A---T---
S-KYVAKI-------ET-----------DGTG--------------KITVTSQG--IKN-
---K--------------------ADGTTG----------------------------

>ur|UPI0006D67964|12_123|1.5e-12|unclassified_Pseudoalteromonas
FTLIELMIVVAIIGILAAVALPAYQDYTLKARFAEVKTSAASVKTTMAQCLQEN------
--N---N-II-ASC----D------SYADL------GIA-APV---------A-------
N-DNLASV-------AIA----------ETTG--------------VITATGTA--SA--
----------GGF----TYTLTPPTVAA------------------------------

>ur|UPI00260D1AEA|7_108|1.7e-12|uncultured_Halomonas_sp.
FTLIEVMIVVAIIGILASIAVPSYQRYVDRAHRTQAKTLMNEMAQRLERRYSQA------
--Y---S-YG-T-VGANQT------PSALNLSSSLEGVP-DDA---------S-------
--RYRFRI-------DITD---------GGNGY-------------VIKA----------

>ur|A0A6L8MPT2|12_116|1.9e-12|Duganella_lactea
FTLIELMIVVAIIGILAAVAIPAYSNYTIKAKMANAQSAVDSLKTAVALCVQEA------
--G---GVLT-D-C----N------TGSN-------GIP-------------A-FTP---
T-KEVKTA-------TV------------AAG--------------VITATFQD--TGV-
---GSG-V--DTL----AV---------------------------------------

>ur|I5B0S1|12_106|2e-12|Desulfobacter_postgatei_2ac9
FTLIELMIVVAIIGILAAIAVPQFASYRMRSYNAAAKAVVHNLKADSANLNSEL------
--G---V-YG------HTE------AAAA-------ALN-AGD---------A-------
GAGAADSV--------------------ADTG--------------LIAAATSG--AAG-

>ur|A0A3S4JDZ8|7_112|2.2e-12|Cedecea_lapagei
FTLIELMVVIGIIAILSAIGIPGYQNYLRKAALTDMLQTFLPYRTAVELCAIER------
--G---G-IT-G-C----N------AGSN-------GIA-ETQ---------T-------
T-RYIRAM-------DV------------SSG--------------IVTLNGQD------
------SL--SGL----TAIFTPQ------------------------------WS---

>ur|A0A932BK51|9_145|2.4e-12|Betaproteobacteria_bacterium
FTLIELMIVVAIIGILAAVALPAYQTYTIKAKVSEAILATSQCRTAVSEQYQTA------
--S---T-GT-S-F----V------GGGW-------GCE-STS---------G-SQA---
T-KYVQSI-------TT-----------DDNG--------------VITVTTSA--AAD-
---LKD-A--NST----TITLTPVRADGTTVIVK----TDAP-TQV-------GQFLCR-
----A--------------------
>ur|UPI0015D2B3AE|8_125|2.7e-12|unclassified_Acinetobacter
FTLIELMIVVAIIGILAAIAIPAYQDYTVRARVSEGLTAASAVKATVTENISNN------
--G---G-AL-A------A------DNCV-------GFT-DTT---------G------G
T-GNVASV-------ACA----------PATG--------------IISVTMGA--KA--
----------QNV----AMTLEPTPSA----------------TG-------VTWVCKS

>ur|A0A9E6V4A8|11_100|2.9e-12|Betaproteobacteria_bacterium
FTLIELMIVIAIIGILAAIAIPMYQDYTVRAKVTEGIAAAAPCKLGVAEFYAAN------
--S---S-YP-G------S------LQSA-------GCT-TVV-----------------
T-TYVSGV-------NV-----------GTGG--------------VVTVTLQ-------

>ur|A0A1V8M703|13_100|3.2e-12|Methyloprofundus_sedimenti
FTLIELMIVVAIIGILASIALPAYQDYIVKANAGAAVGNLGGQKIKVAEAFSLG------
--V---G-ND-G-A-----------PGTL-------GCK-DTG--------NS-------
--------------DIPD---------CGTG--------------GVLSTSVG------

>ur|UPI001B31F50F|7_99|3.5e-12|Aeromonas
FTLIELMIVVAIVAILAAIALPAYQDYVKRAKGAELSSAMASTKTAMEVCAASQ------
--E-----FT-N-C----------------------NYP-TFT---------A-------
T-QFVASV-------SPATF-------TSAAA--------------SIVATGKG------
------DL--

>ur|A0A1M6YEV5|9_158|3.9e-12|Pseudomonas_punonensis
FTLIELMIVVAIIGILAAIALPAYQDYTVRAKVGEAIIAGSAAKGLMSEAFQTD------
--G-----VA-G-M----T------AAAV-------AFN-ASP--------IA-ERT---
S-KYVSNV-------AIAE---------ASPW--------------TITVTLAA--TQAN
G-IPNV-L--NGQ----TLTYSPNVANAVP--------TAAS-VGA-------IDWACGS
----ATTA-------TATGRGLG---
>ur|A0A6I6HQ68|14_106|4.3e-12|Variovorax_paradoxus
FTLIELMIVVAIIGILAAVALPAYQDYIVKSKWAVNITEVEGVKSAVKACMDDS------
--A---G-DG-TLC----D------TLAD---LQKYGYA-GTA--------LT-------
TPKYGSIV-------ALT----------GTAG----------------------------

>ur|UPI0011D55C13|12_128|4.9e-12|Vibrio_cholerae
FTLIELMIVVAIIGVLAAIAVPAYKSYVTKSELATGAATLRSLLTNIDMYHQEK------
--G---T-YA-G-I----S------LPEI-------GAT-TGM-----------------
--SGLGKI-------EVTGT--------VSTA--------------TATFTFDN--SS--
-------V--DKA----VITYAK---------------------SS-------SGWACS-
----I----AD---------------
>ur|UPI0018CD2356|12_114|5.4e-12|unclassified_Pseudoalteromonas
FTLIELMIVVAIIGILAAVALPAYQNYTKKAYFSEVILATAPYKAGVQTCILTN------
--A-----IT-E-C----D------TGVK-------GIP-ATT---------A-------
T-KVVASV-------SV------------TDG--------------VILVTPKA--ING-
-------IA-VGD----TYTL---------------------------------------

>ur|X0QWT9|7_102|6.1e-12|Psychrobacter_sp._JCM_18901
FTLIELMIVIAIIGILAAIALPAYQDYTKRARVGEALSLAGGAKTAVTEFYSSN------
--N---A-WP-A------N------NTSA-------GLP-ATP---------A-DIN---
G-NSVTSV-------TV------------NNG--------------KISVAVKD--NI--

>ur|UPI000E588D1D|8_145|6.7e-12|Neisseria_lactamica
FTLIELMIVIAIVGILAAVALPAYQDYTARAQMSEALTLAEGQKAAVVEYYSDN------
--G---T-FP-A------T------NTSA-------GIA-PAS---------T--IT---
G-KYVKSV-------NVSGT--------ATSA--------------VITSTMKS--SGV-
---NKD-I--SGK----TLLLQGKQNS-----------------GS-------FEWTCV-
----KGTV-DD----KFLPSSC----
>ur|A0A1G9QKR8|7_122|7.4e-12|Halomonas_muralis
FTLIELLLAMVIIGILAAIAIPAYQGYVERSYRADAQAELLEIAQRLERRYSQT------
--F---S-YA-N-A----------------------GGS-ASS---------A-------
--VQVGRV--------------------PEGG----------DARYLIILTMAN------
----------SGS----SYTLNAAPQG-----------------TQ-------QSDECG-
----TLTL-NQ---------------
>ur|A0A1L1PTG0|9_116|8.3e-12|Hydrogenophaga_intermedia
FTLIELMIVVAIIGILAAVALPAYQDYTVRARVSEGLVGASAAKVNVQDVLSSG------
--N---P-GDDA------A------GYSL-------GFR-EVS---------A--ANGNQ
T-RNVEAL-------SIA----------AATG--------------VITVDMQA--AA--
----------GGG----TLII--------------------------------------

>ur|A0A1J5IEK2|13_124|9.1e-12|Syntrophaceae_bacterium_CG2_30_58_14
FTLIELMIVIAIIGILAAIAIPQFTAYKKRGYNTEAKSDAKNAYTAAQAYFSDT------
--P---G-EAVN----AGS------LTAY-------GYT-TSP-----------------
---------------HVTFN--------VQDG-----------SQAALGMTAVG--SAR-
----------GGT---GDVTYSVN--------------------SA--------------
-----GTI------------------
>ur|A0A1I2K2S9|11_100|1e-11|Fontimonas_thermophila
FTLIELMIVVAIIGILAAIAIPAYQDYTIRSQVAEGLSLASGAKAAVWDFMSNT------
--G---R-TP-A------T------NASA-------GLP-NAA---------S--IT---
G-NYVASV-------TV------------TNG--------------VIEVA---------

>ur|A0A157ZI39|30_148|1.1e-11|Caballeronia_fortuita
FTLIELMIVLAIVGVVAAYAIPAYQDYLARSRVGEGIALAAGARLAVAENAASG------
--A---D-LA-G------------------------GFA-SPP---------G-------
T-RNVESV-------HVD----------SDNG--------------QITIAYST--RVAP
-------A--GSN----TLVLVPSTPDNADTPT--------------------------
----SRVALSR---------------
>ur|UPI0024897D43|8_111|1.2e-11|Methylotenera_sp.
FTLIELMIVVAIIGILAAVAIPSYQNYTKKARFSEVLSLSDTFKQSVGICITDN------
--TANPGTAT-G-C----S------NGLQ-------GVE-AAP---------A-A-----
T-PNVASL-------VT------------ANG--------------IITGTGTT--AA--
----------GGY----TS--

>ur|UPI002589FF2C|9_120|1.4e-11|uncultured_Alcanivorax_sp.
FTLIELMIVVAIIGILAAVALPAYQDYTVRAKVSEGILAGSSCRTTVTEVYQSG------
--S---A-LP-A-------------ANAW-------GCE-TSA---------ALGNN---

T-AKVDSV-------AT-----------GDAG--------------AITITMNT--TEIA
--QLGA-TG-AGN----IITL--

>ur|Q462L9|1_98|1.5e-11|Neisseria_mucosa
-TLIELMIVVAIIGILAAIALPAYQDYTARAQISEAITLSDGQKAAVTEYYADK------
--G---A-FP-T------S------NAEA-------GVA-SAS---------T--IK---
G-KYVTSV-------TI-----------GANG--------------VITALMNS--SGV-
---AD---

>ur|A0A1H1XYK1|8_143|1.7e-11|Halopseudomonas_litoralis
FTLIELMIVVAIIGILAAIALPAYQDYTVRAKMSEVIGFASSARTAVSECAVAE------
--G---G-LA-N-C----G------DGAS-------GVD-VTE---------M-AAA---
S-DYLTSV-------AI------------AAG--------------IITLELDW--SEL-
---GGS----GTG----DLVYTPTYSA-----------------GG-------VAWECGM
----SVAD-DY----KYVPNAC----
>ur|A0A0P7DYQ8|9_118|1.9e-11|Pseudoalteromonas_lipolytica
FTLIELMIVVAIIGILAAVALPAYSNYQATSKLTAGLAEISAGKTGFEIAKNSG------
--T---A-TP-T-------------LADA-------GLS-ATT-----------------
--TNCN-I-------AVDAT--------------------------SIACTIQN--APTQ
-------I--SGA----VLTWTRS--------------------DD-------GDWSCA-

>ur|UPI002494D8B2|9_124|2e-11|Pseudoalteromonas
FTLIELMIVIAIIGILAAVALPQYSDYTNRAKATEVMLAASTVKTCASEKAQV-------
--G---A-SP-A------D------CSAD-----------FKA---------T-------
--KYVSAV-------AI-----------TTTG--------------AITATAAD--DL--
----------LGL----TIVLTPQNGDNAATAANFTAGFTI-------------------

>ur|UPI001431545B|7_98|2.2e-11|Aeromonas
FTLIELMIVVAIVAILAAVALPAYQNYTKKAKATELTSAIGQVKTELEVCAQTS------
--S-----LP---C----------------------NAS-AAA-----------------
S-KFVAGV-------SGAI---------ASSG--------------VATITGQG--ASD-
-------IS--

>ur|UPI0007D84925|16_125|2.5e-11|Ralstonia_solanacearum
FTLIELMIVVAIIGILAAIAIPAYQDYTVRSRVVEGLELAANAKALVVENAANG------
--Q---S------------------SLAQ-------GAS-TLP---------A-------
D-RNVSAL-------DIN----------STTG--------------EITVAYTS--AVAG
-------T--GAN----TLVLTPYSGNSSA-----------------------------

>ur|UPI00254E1BA5|7_124|2.8e-11|Acinetobacter_radioresistens
FTLIELMIVVAIIGILAAIAIPAYQNYTKRAHVTEGLNLAGGAKAAVTEYFSSK------
--G---E-WP-A------N------NTAA-------GIA-KDV---------S--IK---
G-NAVKSV-------TV------------DAS--------------KITIKYNT--KVE-
----------NDK----TIILQGASSV-----------------GG-------VTWTCK-
----N--------------------
>ur|A0A3E1RB59|8_121|3.1e-11|Rhodoferax_lacus
FTLIELMIVVAIIGILAAVALPAYQTYTNKAKFSEVILATAAHKIAIELCLQDQ------

```
--G---A-VG-ANC----A------AGSN-------GVP-VDI---------T--TA---
S-GRVASV-------TTD----------ATTN--------------AVTATAVA--TGG-
-------L--GGQ----TYILNPAITG--------------------------------
-------------------------
>ur|UPI00272E5D33|12_139|3.5e-11|Methylobacter_sp._BlB1
FTLIELMIVVAIIGILAAIAIPAYNDYTAKAQASEAFELIDGLKSPVTAAYSEV------
--G---T-WTIP----------------------------AGS-----------VIK---
G-KYVDKI-------EAAGA--------DKAW--------------TLKATYVA--SGA-
---NTK-V--IGK----TVTFTYD-------------------NST-------GQWTCA-
-----SDL-DA----AVKPASC----
>ur|A0A244CTR0|8_125|3.9e-11|Pseudoalteromonas_ulvae
FTLVELMIVIAIIGILAAVALPEYQGYVAKSDLSSCHKEIHSGIVLFEIKVNSG------
--T---P-PS-S----ASN------LSEI-------NVR------------KA-------
--SSCASH-------AMTAN-------------------------TISGIVKG--SAP-
-------A--AGA----KIELIRD-------------------LNT-------GVWSCE-
----VTSR---------------P--
>ur|A0A1D8IRI0|9_139|4.3e-11|Acidihalobacter_yilgarnensis
FTLIELMIVVAIIGILAAIAIPAYQDYTIRAKVTEGLNLAGAAKSAIWDTYGNL------
--G---T-FP-T-V---GG------NASY-------GLP-ATI---------A-------
G-TNTSNV-------TI-----------GSQG--------------LITITYKN--NL--
---GGA-A--NGK----TLLLQPWTHG-----------------GV-------IEWICY-
----AANK------PAQTP------L
>ur|A0A212Q699|13_101|4.8e-11|Marinobacter_sp._es.042
FTLIELMIVVAIIGILAAIAIPAYQDYVEQSRVDSCLAELKAQTNNWLIEYSQD------
--P---SNLS-T------------------------AVQ---------------------
--GACASI------------------SAPASG------------------------SAP-
-------T--NGS----------------------------G-VPT-------ANW----
-------------------------
>ur|UPI0012439C37|15_144|5.3e-11|Cysteiniphilum_sp._JM-1
FSLIELLIVIAIIAILTAIAIPMYTSYTERAKTAEAYSFIGSDKTYVAEQINSK------
--G---I-TS-G----TIS-----------------GLA-TTGG------TKT-------
G-KYGGVV-------------------SNAAG--------------VITYKFTG--AAG-
-----T-L--SGN----TVFFTPSVGS-----------------AG-------VTWACS-
----STGT-------SFSADPCD---
>ur|A0A522FH80|10_126|6e-11|Legionella_sp.
FTLIELMIVVAIVGILAAIAIPAYQDYTIRARVTEGLSLASSAKLAVSETTLAN------
--N---A-LP-A------T------QAAT-------GYT-SPA---------A-------
T-TNVTSI-------AI-----------GANG--------------VITVTYTA--AA--
----------GGG----TIIMTPTLTA----------------NGD-------LTWVCTG
-------------------------
>ur|A0A5P9EM50|13_131|6.6e-11|Marinobacter_sp._THAF39
FTLIELMIVVAIIGILAAVAIPAYQDYTIRAKVSEVLTVASAARTSVAEYYIST------
--G---S-MP-A------T------TTAA-------GIN-TDT---------G------Q
S-DYITNI-------AFAT---------SSTGT-------------QLTYTLGN------
--LGGT-A--DAS----TFEYLVTGNNNGVQV---------------------------
-------------------------
>ur|UPI0007828AA3|14_144|7.5e-11|Variovorax_boronicumulans
```

FTLIELMIVVAIIGILAAVALPAYQDYTTRAKVSELLLMAAPAKLAVAETASSF------
--G---D-LQ-S-V----T------ATNN-------GYS-FPG---------A-------
T-KYVSGV-------TI-----------AAGG--------------EVTVASLI--PG--
----------AGG----NIVLTPTAVATG--------------SAQ-------LTWKCT-
-----TTI-NV----KFVPAEC----
>ur|UPI001B82FFE3|15_114|8.3e-11|Massilia_sp._ZL223
FTLIELMIVVAIIGILAAVAIPAYQSYTVKAKIGAALGSVSSIKTAVGVCAQEN------
--G---GTLD-N-C----D------SGAS-------GIV-AEA---------D-FVA---
T-KEVAGV-------AV------------ADG--------------VLTVTLGT--GIAT

>ur|A0A1V3JUQ7|13_103|9.5e-11|Rodentibacter_myodis
FTLIELMIVIAIIAILATIAIPSYQNYTKKAAMSELLQAASPFKSDVELCIYGT------
--G---N-AA-N-C----S------SGSN-------GVA-TDI---------T--TP---
K-GYVKSI-------TT------------NAG--------------VITVV---------

>ur|A0A1X7AH56|8_120|1.1e-10|Parendozoicomonas_haliclonae
FSLVELMIVVAIIGILSAVAYPSYQNYVTRNACEDARGTLLAAASALERYRSEN------
--R---G-YT-------------------------GAV-AGTHFP----DRSPVD----
--GGTQNY-------TIAFD--------TAPT--------------AITFTLTA--TSRA
---G---S--NYP----AITLNQSG----------------------------------

>ur|A0A0U5IL65|8_104|1.2e-10|Thiocapsa_sp._KS1
FTLIELMIVVAIIGILAAIALPAYQDYTMRARVSELVLAASAARTCVTESSQLA------
--G---A------------------ASGA-------GCE-VGK-----------------
T-DLVSTA-------VV-----------STAG--------------KIVITGVA--SA--
----------GST----VITLT-------------------------------------

>ur|A0A846HRX6|12_108|1.4e-10|Halochromatium_sp.
FTLIELMITVAIVGILAAIAYPSYQNSLQRSWRSNAASCLMELAQQMERRFTSS------
--S---S-YL-------------------------GAL--------------------
--PAVGCI---------------------NEG----------SMPTRYTFSFAA--SEPN
----------ANT----FVILAQP-----------------------------------
------------------------V
>ur|UPI0028835066|9_108|1.6e-10|Thiothrix_winogradskyi
FSLIELMIVVAIIGILAAVALPAYQDYTIRSRIMEGIRLANPAKAAVANHAASV------
--A---D-IAVA----ATD--WNNQENNN-------GTV-ATS-----------------
--KYVEKI-------HID----------NPTG--------------TITITFNS--ST--

>ur|A0A3A3GD59|8_103|1.8e-10|Noviherbaspirillum_sedimenti
FTLIELMIVVAIIGILAAVALPAYQDYTVRAKMSEVVLAASSCRTSVTETRQTTTN----
-IA---S-IP-A-------------AGAW-------GCE-SSV---------P---T---
T-KYVASI-------AT-----------NTTG--------------AIEVRVNS--T---


```
>ur|A0A4R2IDH6|8_102|2e-10|Dokdonella_fugitiva
FTLIELMIVVAIIAILAAIALPAYQDYMVRAKLSEVFVAGDACKNSVVEFYEGQ------
--G---A-LP-G------N------LTSA-------GCN-NNK---------T-------
--KYVADT-------AV------------NAG--------------VITIKTSG--AVD-
---LG-------------------------------------------------------
-------------------------
>ur|UPI0019257E6E|7_96|2.3e-10|Enterobacteriaceae
FTLIELMVVIGIIAILSAIGIPAYQSYLRKAALTDMLQTFTPYRTAVELCAIDR------
--G---G-VT-Q-C----N------AGSN-------SIP-AVR---------T-------
T-RYVSSM-------TV------------NQG--------------VIALTG--------
------------------------------------------------------------
-------------------------
>ur|A0A8B6S1Z6|7_115|2.7e-10|Aeromonadaceae
FTLIELMIVVAIVAILAAIALPAYQSYTQKARFTEVVSSAGAVKTALEVCFQTT------
--T---D-VT-T-C----A------ATANA------TVSGAYP---------S-------
--GLVESV------------------VASVSG-----------SVPTITATGTT------
----------SGAP--GTYIMTGA------------------------------------
-------------------------
>ur|I2FFI8|7_112|3.1e-10|Pseudomonas_syringae_group
FTLIELMIVVAIIGILAAVAIPSYNTYTLKAKVSEASAISAPAQQALALAFNDG------
------S-LS-A-A---TT------NASL-------GLP-AAA---------S--IT---
S-KYVASV-------TAVG-------TSATQG--------------TVTVVMQA--TG--
---SAD-V--D-------------------------------------------------
-------------------------
>ur|A0A9D7AFW2|7_111|3.6e-10|Budviciaceae
FTLFELMIAIAIIAILTAIGLPAYQGYIQKAALTDMLQAMVPYKTAVELCALES------
--G---E-LS-Q-C----N------NGNA-------GIA-ATK---------E-------
T-RYVSRI-------AV------------ANG--------------VISLAGKN------
------AL--SGL----SVDLTPTL-----------------------------------
-------------------------
>ur|UPI00278063F0|4_104|4.1e-10|Variovorax_boronicumulans
FTLIEVMITVAIIGILAAVALPSYRDYVLRGRLVDAANALSIAKADMERYFQDN------
--R---T-FV--------------------------GAG-ACT---------S-------
--STVGSF-------TISCV------GTPTAT--------------TYSLQAVG--SGA-
-------A--NGF----TFTI---------------------------------------
-------------------------
>ur|UPI00070E4690|7_107|4.9e-10|Xanthomonadaceae
FTLIELMIVVAIIAILAAIALPAYSDYTKKSKVSEVVLAASALRTAVAEYASSN------
--N---T-LP-G-------------ASWS-------GFE-AQS-----------------
S-KYVSGV-------TWDGT--------------------------KITATAQS------
--IGSG-V--DGA----TIEL---------------------------------------
-------------------------
>ur|UPI001BAC2B37|12_108|5.8e-10|Kistimonas_asteriae
FTLIELMIVVAIIGILSAVAIPAYQDYTKRAHVSEGLNLASGVKAAIAEYYATE------
--G---A-WP-L------N------NASA-------GLD-ASL---------A-------
G-NAVTGV-------VV------------SND--------------TITITYNT--KVD-
----------AGG-----------------------------------------------
```

>ur|A0A410UR01|10_113|6.9e-10|Janthinobacterium_sp._17J80-10
FTLVELMITVAIIGILASIALPSYRNYLVRGRIPDATAGLAAKRVRMEQFFQDN------
--R---T-YV--------------------------GAP-DCA---------S---D---
A-NTSKYY-------TFSCD------GAATAN--------------AFILQAVG--AGP-
-------M--TGF----KFTVD--------------------------------------
