# Supplementary material for: Structure of the Pseudomonas aeruginosa PAO1 Type IV pilus
Source: PLoS Pathog. 2024 Dec 12;20(12):e1012773. doi: 10.1371/journal.ppat.1012773 (PMC11670995; doi:10.1371/journal.ppat.1012773)
Supplement: S2 File — (DOCX) [file ppat.1012773.s017.docx]

| **Chain** | **Resid. no.** | **Resid. type** | **Interact. chain** | **Resid. no.** | **Resid. type** |
| --- | --- | --- | --- | --- | --- |
| M | 11 | GLU | L | 9 | LEU |
| M | 15 | VAL | L | 12 | LEU |
| M | 15 | VAL | L | 9 | LEU |
| M | 18 | ILE | L | 12 | LEU |
| M | 18 | ILE | L | 9 | LEU |
| M | 19 | ILE | L | 12 | LEU |
| M | 19 | ILE | L | 7 | PHE |
| M | 22 | LEU | L | 19 | ILE |
| M | 22 | LEU | L | 16 | VAL |
| M | 22 | LEU | L | 15 | VAL |
| M | 131 | VAL | L | 70 | THR |
| M | 131 | VAL | L | 81 | GLY |
| M | 145 | LYS | L | 88 | LYS |
| M | 145 | LYS | L | 89 | LEU |
| M | 145 | LYS | L | 112 | THR |
| M | 146 | GLY | L | 88 | LYS |
| M | 146 | GLY | L | 86 | ALA |
| M | 148 | ASP | L | 88 | LYS |
| M | 22 | LEU | K | 7 | PHE |
| M | 27 | ILE | J | 10 | ILE |
| M | 30 | TYR | J | 14 | ILE |
| M | 30 | TYR | J | 11 | GLU |
| M | 30 | TYR | J | 10 | ILE |
| M | 33 | TYR | J | 18 | ILE |
| M | 34 | VAL | J | 21 | ILE |
| M | 34 | VAL | J | 14 | ILE |
| M | 37 | SER | J | 21 | ILE |
| M | 37 | SER | J | 18 | ILE |
| M | 38 | GLU | J | 21 | ILE |
| M | 41 | SER | J | 25 | ILE |
| M | 41 | SER | J | 21 | ILE |
| M | 44 | ALA | J | 25 | ILE |
| M | 45 | THR | J | 25 | ILE |
| M | 45 | THR | J | 26 | ALA |
| M | 48 | PRO | J | 28 | PRO |
| M | 48 | PRO | J | 30 | TYR |
| M | 51 | THR | J | 33 | TYR |
| M | 52 | THR | J | 33 | TYR |
| M | 55 | GLU | J | 33 | TYR |
| M | 55 | GLU | J | 36 | ARG |
| M | 59 | ARG | J | 145 | LYS |
| M | 59 | ARG | J | 36 | ARG |
| M | 73 | SER | J | 141 | MET |
| M | 73 | SER | J | 32 | ASN |
| M | 80 | VAL | J | 28 | PRO |
| M | 80 | VAL | J | 29 | GLN |
| M | 82 | VAL | J | 26 | ALA |
| M | 82 | VAL | J | 27 | ILE |
| M | 82 | VAL | J | 28 | PRO |
| M | 86 | ALA | J | 24 | ALA |
| M | 86 | ALA | J | 26 | ALA |
| M | 87 | ASN | J | 24 | ALA |
| M | 30 | TYR | I | 9 | LEU |
| M | 33 | TYR | I | 13 | MET |
| M | 33 | TYR | I | 9 | LEU |
| M | 47 | ASN | I | 23 | ALA |
| M | 50 | LYS | I | 27 | ILE |
| M | 57 | LEU | I | 114 | SER |
| M | 57 | LEU | I | 115 | PRO |
| M | 58 | SER | I | 38 | GLU |
| M | 58 | SER | I | 34 | VAL |
| M | 59 | ARG | I | 111 | GLY |
| M | 59 | ARG | I | 112 | THR |
| M | 60 | GLY | I | 113 | SER |
| M | 60 | GLY | I | 111 | GLY |
| M | 60 | GLY | I | 112 | THR |
| M | 126 | ARG | I | 31 | GLN |
| M | 128 | ALA | I | 31 | GLN |
| M | 128 | ALA | I | 116 | LYS |
| M | 129 | ASP | I | 31 | GLN |
| M | 129 | ASP | I | 29 | GLN |
| M | 130 | GLY | I | 29 | GLN |
| M | 131 | VAL | I | 29 | GLN |
| M | 145 | LYS | I | 24 | ALA |
| M | 146 | GLY | I | 24 | ALA |
| M | 7 | PHE | N | 19 | ILE |
| M | 9 | LEU | N | 15 | VAL |
| M | 9 | LEU | N | 18 | ILE |
| M | 9 | LEU | N | 11 | GLU |
| M | 12 | LEU | N | 18 | ILE |
| M | 12 | LEU | N | 19 | ILE |
| M | 12 | LEU | N | 15 | VAL |
| M | 15 | VAL | N | 22 | LEU |
| M | 16 | VAL | N | 22 | LEU |
| M | 19 | ILE | N | 22 | LEU |
| M | 70 | THR | N | 131 | VAL |
| M | 81 | GLY | N | 131 | VAL |
| M | 86 | ALA | N | 146 | GLY |
| M | 88 | LYS | N | 145 | LYS |
| M | 88 | LYS | N | 146 | GLY |
| M | 88 | LYS | N | 148 | ASP |
| M | 89 | LEU | N | 145 | LYS |
| M | 112 | THR | N | 145 | LYS |
| M | 7 | PHE | O | 22 | LEU |
| M | 10 | ILE | P | 30 | TYR |
| M | 11 | GLU | P | 30 | TYR |
| M | 14 | ILE | P | 30 | TYR |
| M | 14 | ILE | P | 34 | VAL |
| M | 18 | ILE | P | 37 | SER |
| M | 21 | ILE | P | 41 | SER |
| M | 21 | ILE | P | 37 | SER |
| M | 21 | ILE | P | 38 | GLU |
| M | 21 | ILE | P | 34 | VAL |
| M | 24 | ALA | P | 87 | ASN |
| M | 25 | ILE | P | 45 | THR |
| M | 25 | ILE | P | 41 | SER |
| M | 26 | ALA | P | 45 | THR |
| M | 26 | ALA | P | 82 | VAL |
| M | 26 | ALA | P | 86 | ALA |
| M | 27 | ILE | P | 82 | VAL |
| M | 28 | PRO | P | 80 | VAL |
| M | 28 | PRO | P | 82 | VAL |
| M | 28 | PRO | P | 48 | PRO |
| M | 29 | GLN | P | 80 | VAL |
| M | 30 | TYR | P | 48 | PRO |
| M | 32 | ASN | P | 73 | SER |
| M | 33 | TYR | P | 55 | GLU |
| M | 33 | TYR | P | 52 | THR |
| M | 36 | ARG | P | 55 | GLU |
| M | 36 | ARG | P | 59 | ARG |
| M | 141 | MET | P | 73 | SER |
| M | 145 | LYS | P | 59 | ARG |
| M | 9 | LEU | Q | 30 | TYR |
| M | 9 | LEU | Q | 33 | TYR |
| M | 23 | ALA | Q | 47 | ASN |
| M | 24 | ALA | Q | 145 | LYS |
| M | 24 | ALA | Q | 146 | GLY |
| M | 27 | ILE | Q | 50 | LYS |
| M | 29 | GLN | Q | 129 | ASP |
| M | 29 | GLN | Q | 130 | GLY |
| M | 29 | GLN | Q | 131 | VAL |
| M | 31 | GLN | Q | 129 | ASP |
| M | 31 | GLN | Q | 128 | ALA |
| M | 31 | GLN | Q | 126 | ARG |
| M | 34 | VAL | Q | 58 | SER |
| M | 38 | GLU | Q | 58 | SER |
| M | 111 | GLY | Q | 60 | GLY |
| M | 111 | GLY | Q | 59 | ARG |
| M | 112 | THR | Q | 60 | GLY |
| M | 112 | THR | Q | 59 | ARG |
| M | 113 | SER | Q | 60 | GLY |
| M | 114 | SER | Q | 57 | LEU |
| M | 115 | PRO | Q | 57 | LEU |
| M | 116 | LYS | Q | 128 | ALA |
