# Supplementary material for: Structure of the Pseudomonas aeruginosa PAO1 Type IV pilus
Source: PLoS Pathog. 2024 Dec 12;20(12):e1012773. doi: 10.1371/journal.ppat.1012773 (PMC11670995; doi:10.1371/journal.ppat.1012773)
Supplement: Attachment — Submitted filename: 146161_1_rebuttal_3071833_sg1t7h.pdf [file ppat.1012773.s018.pdf]

Full Revision

Manuscript number: RC- 2024-02464

Corresponding author(s): Tanmay Bharat

[Please use this template <u>only</u> if the submitted manuscript should be considered by the affiliate journal as a full revision in response to the points raised by the reviewers.

If you wish to submit a preliminary revision with a revision plan, please use our "[Revision Plan]" template. ***It is important to use the appropriate template to clearly inform the editors of your intentions.****]*

1. General Statements [optional]

The goal of the study was to report the structure of the *Pseudomonas aeruginosa* Type IV pilus. We have solved a 3.2 Å-resolution cryo-EM structure of the pilus, discussed implications about the biology arising from our structure and compared our resolved details to other Type IV pilus structures from different organisms. We have addressed all concerns raised by the reviewers in the revised version except for one request for additional functional experiments.

We would also like to highlight that a competing study reporting the same structure (at a lower resolution) was published concurrently with the submission of our manuscript (Thongchol *et al*, Science 2024). Considering this and the goal of our paper discussed above, we feel that detailed functional analyses are beyond the scope of this straightforward manuscript reporting the structure of the *P. aeruginosa* Type IV pilus. We have proactively engaged with the results of the other manuscript as suggested by Reviewer #3, who correctly points out that our structure should be of high interest to microbiologists, because it is the highest resolution structure of *P. aeruginosa* Type IV pilus.

2. Point-by-point description of the revisions

This section is mandatory. Please insert a point-by-point reply describing the revisions that were <u>already carried out and included</u> in the transferred manuscript.

Reviewer #1

Summary
The study titled "Structure of the Pseudomonas aeruginosa PAO1 Type IV pilus" by Ochner and colleagues utilised cryo-electron microscopy (cryo-EM) to determine and describe the atomic structure of a complete type IV pilus (T4P) filament from Pseudomonas aeruginosa in its native state at an impressive resolution of 3.2 Å. The authors use state-of-the-art cryo-EM methodology, and the detailed description of their procedures allows for an adequate replication of the results.

The T4P are essential for the virulence of P. aeruginosa, which is a clinically important human pathogen, as they play a crucial role in biofilm formation, a major factor in its resistance to antibiotics and ability to cause infections. Therefore, understanding the molecular mechanisms behind the ability of these bacteria to establish infections is vital, and this high-resolution structure of T4P provides valuable insight into this process.
Overall, this reviewer acknowledges the importance of the structure presented here and its significance to our understanding of P. aeruginosa infection and persistence, and hence is positive about the publication of the results, however, at its current state the manuscript raises the major questions outlined below, which must be addressed and corrected.

We thank Reviewer #1 for their constructive comments, which we address point-by-point below:

Major comments
The authors propose a model where T4P interact with the type IV secretion systems (T4SS) in the P. aeruginosa membrane. However, there is no current evidence in the literature to support a direct interaction between T4P and T4SS as these are functional and structural distinct secretion systems. T4P biogenesis is mediated by a specialised secretion complex (homologous to type II secretion systems), spanning both bacterial membranes and consisting of the outer membrane secretin subcomplex, the alignment subcomplex, and the inner membrane motor complex. This reviewer recommends that authors refer to the comprehensive review by Hospenthal and colleagues [PMID: 28496159] that details the T4P biogenesis and Craig and colleagues [PMID: 30988511] that provides an in-depth analysis of T4P secretin architecture. This reviewer recommends the authors to remove any misleading claims regarding a T4P-T4SS interaction. Furthermore, the introduction would benefit from a brief overview of the T4P biogenesis and secretin architecture to prevent any further confusion.

We thank Reviewer #1 for raising this and apologise for the incorrect labelling. We have corrected the text and figures as requested and have updated the introduction to include a paragraph summarising T4P biogenesis (L46-51, 66-71):

"T4P biogenesis employs a multi-component membrane complex to transport pilin subunits into the growing pilus, extruding the pilus across the periplasm and through an outer membrane secretin channel for surface display[14,15]. T4Ps are dynamic filaments that can extend and retract[16,17], allowing cells to rapidly respond to a changing environment, particularly when encountering a favourable surface suitable for colonisation." (L46-51)
and
"Other studies have elucidated the organization of the secretion machinery of T4Ps in different bacterial species using electron cryotomography (cryoET), which features ring-like densities corresponding to the secretin channel, assembly and retraction complexes[32–34]. Cryo-EM analysis of the *P. aeruginosa* secretin, a homomultimer of PilQ, revealed a channel with a gate at the periplasmic face, resembling the shape of secretins involved in type II and type III secretion[35]." (L66-71)

While this study offers a higher resolution structure of P. aeruginosa T4P (3.2 Å) compared to the previously described work on the P. aeruginosa T4P (8 Å) described by Wang and colleagues [PMID: 28877506], the manuscript fails to convey the significance of this improvement. The authors should directly compare the new structure with the previously obtained cryo-EM structure, similarly to how they tackled the comparison to the X-ray crystallography structure (Figure S6). A dedicated figure visualising the key differences and benefits associated with the higher resolution is necessary to highlight the manuscript's significance.

To address this, we have added a supplementary figure (Fig. S5), in which we compare our structure with the structure of the PAK strain pilus published by Wang et al. (Structure 25, 1423-1435.e4). We compare both the EM density maps and the resulting models, highlighting that the improved resolution of our map allows direct side chain modelling as the corresponding densities are resolved in the 3.2 Å map.

Furthermore, the authors should specify that the reported T4P belongs to the type IVa category and the "globular domain" of PilA should be further differentiated into the αβ loop and D region - widely accepted motifs present in the structures of type IV pilins [PMID: 31784891]. Highlighting them is crucial due to their roles in receptor binding, microcolony formation, and antigenic variation, warranting their inclusion in the manuscript.

We have marked the αβ-loop and D-region in Fig. 2c and have changed the corresponding text accordingly, highlighting these regions, and identifying the reported T4P as belonging to the type IVa category. We have also included the citation referenced by the reviewer (L132-140):

“Two additional structural motifs common to pilins can be identified within the globular domain, namely the αβ-loop linking the α-helix and the β-sheet and the D-region at the C-terminus[41,42] (marked in Fig. 2c). The innermost part of the T4P is stabilised by hydrophobic stacking of the N-terminal α-helices of PilA, containing a row of hydrophobic residues (amino acid residues 7-22, where residue 7 is the N-terminus of the mature pilin, consistent with the signal sequence length expected for a Type IVa pilin[43,44]), forming the core of the T4P (Fig. 2d).”

A more detailed display of intersubunit interactions, including the types and numbers of interactions is also recommended, however optional.

In our figure, we aim to display the key hydrophobic interactions within the pilus helical segment by calculating the distances between hydrophobic residues and displaying all pairs of hydrophobic residues whose distance was below a cutoff value of 4 Å (Fig. 2). We have now included a list of all pairs of residues within a distance of 4 Å as a supplementary file (Full list of interacting residues.pdf), which would help the readers use the structural data for future experiments.

Previous studies [PMID: 27698424, PMID: 28609682] hypothesize that disordered loops might be involved in significant T4P stretching, the authors should address how the lack of these structures in their model might affect the filament dynamics.

The T4P stretching discussed in the studies referenced by Reviewer #1 is an interesting point. According to the studies cited by the reviewer (PMID: 27698424, PMID: 28609682), it appears that the disordered parts implicated in this stretching motion are the residues in the melted segment connecting the N-terminal helix to the helical part of the globular domain in the pilus core rather than the disordered loops on the outer pilus surface. As all bacterial pili discussed in this paper feature a melted segment between the two helices, we added a short discussion regarding the role of the melted segment in T4P stretching, citing the studies pointed out by the reviewer (L173-175):
"Several studies have indicated that the melted segments allow the T4P to considerably stretch when subjected to force, thus suggesting a functional role of this structural feature[42,51]."

Lastly, the study lacks experimental validation of the structure, either within the study or referenced from the existing literature and very weakly connects the structure to T4P's biological functions, such as twitching motility or DNA acquisition. For instance, a comparison could be drawn between the surface charge of the pili and its DNA binding capacity.

In response to the critique, we have added all the citations suggested by the reviewer in our answers to previous points (see above). Furthermore, we have added a Supplementary Figure (Fig. S10) showing the surface charge distributions of the T4Ps compared in the manuscript and added a discussion of those results in the context of previous studies regarding the DNA-binding capacity of T4P (Fig S10, L202-208):

"A comparison of surface electrostatics and hydrophobicity properties across the T4P species (Fig. S10 and Fig. S11) shows that all T4Ps feature a hydrophilic outer surface and a hydrophobic core. While the surface charge is predominantly negative or neutral, all T4Ps feature regions of positive charge of varying size (Fig. S10). As patches of positive charge may be related to the DNA binding capability of T4Ps reported in previous studies[54,55], the differences across species might indicate some variation in DNA binding capacity in the different species."

Additionally, the T4P secretin complex of P. aeruginosa documented in [PMID: 27705815] should be modelled alongside the obtained T4P structure to compare the structure diameter with the PilQ secretin lumen.

We have modelled the PAO1 T4P alongside the PilQ secretin structure (Fig. S7), demonstrating that it fits well into the PilQ lumen, and added a corresponding sentence to the main text (L149-151):

"Finally, modelling the *P. aeruginosa* PAO1 T4P alongside the PilQ secretin EM density[35] (Fig. S7) shows that the T4P fits well into the PilQ lumen, which is consistent with the role of the secretin in T4P assembly."

These revisions will strengthen the manuscript by addressing crucial points and highlighting the significance of the high-resolution T4P structure.

Thank you very much for your valuable feedback.

Minor comments

Figure 2 For consistency, the colours of PilA subunits between panels (a) and (d) should match.

We have updated Fig. 2 accordingly.

Figure 3 For clarity, pilins should be coloured by domain.

We have updated Figure 3 as requested.

L41 The word "surfaces" or "target receptors" rather than just "substrates" would be more accurate.
L87 Rather than "other bacteria" consider using "wild-type strains".

We have updated the text accordingly (L41 and L95).

L145-147 For clarity, residues C134 and C147 that form a disulfide bond in the C-terminal loop should be displayed in the figure.

We have marked the C134 and C147 residues in Fig. S8.

L371 For consistency, "h" should be in brackets, following the authors' style.

We have updated the caption to match the style.

Reviewer #1 (Significance (Required)):

General assessment:
The significance of the study stems from a resolution improvement from the previously reported

type IV pilus of P. aeruginosa by Wang and colleagues [PMID: 28877506] and complements well the X-ray crystallography data obtained previously by Craig and colleagues [PMID: 12769840]. Due to the role of T4P in the virulence of P. aeruginosa, the structure provides important biological information about the molecular mechanism of its niche establishment. Moreover, the structure can be used in subsequent drug design against P. aeruginosa infections.

Nature of advance:
The nature of the advance provided by this study is in the added structural detail of the T4P due to the obtained higher resolution of the map. Usually, the highest resolution structure is used to derive the conclusions about the biological functions of the filament, hence the structure provided here will be referenced as the final P. aeruginosa T4P structure in further studies.

Audience:
The higher resolution structure compared to the previously described will be interesting to the translational/clinical drug discovery audiences, which require a high-resolution structure for accurate drug design.

Field of expertise:
Type IV secretion systems, bacterial conjugation, conjugative pili.

Reviewer #2 (Evidence, reproducibility and clarity (Required)):

The manuscript by Ochner et al. reports the cryo-EM structure of the Type 4 pili of Pseudomonas aeruginosa at decent 3.2 resolution with fully resolved pilin fold. It is a straightforward report, using state-of-the-art microscopy and data processing approaches as usual for the group and the figures and data representation are clear. The main findings of the work is that it visualizes the assembled pilus of an important pathogen (Pseudomonas aeruginosa is one of the ESKAPE pathogens with particularly impressive adaptability and Type IV pili are important for substrate colonization and biofilm formation). The PilA pilin fold is not far from that of a previously crystallized isolated homolog from the PAK strain (core hydrophobic N-helix, globular b-sheet-containing exposed CTD) and presents a central melting of the core helix also observed among multiple other PilA homologs from solved G- pilin structures. The main difference for the PAO1 pilus is the tight packing in a significantly thinner filament, which lacks protruding loops or other secondary structure insertions in the core pilin fold. The authors propose that this could lead to increased stability such as to proteases.

Again, the study is quite straightforward and besides the standard and well-executed EM workflow, it uses the classical approaches for pilus overexpression and purification (a PilT mutant that cannot retract and presents more T4P; well established mechanical shearing

protocol for surface release, etc.). The structure is at decent resolution allowing full backbone tracing and side-chain resolution for confident model building, etc.

We thank Reviewer #2 for their comments and address their concerns point-by-point below.

The figures are clear, even if I would encourage some more vivid or at least contrasting colors for the cartoon model in Fig. 2 and some more detailed surface and conservation analyses, especially in terms of packing and surface exposure.

We have updated the colour scheme for the cartoon model in Fig. 2 and have included supplementary figures (Figs. S10, S11) and analysis of surface charges and hydrophobicity of the different T4P species (L202-208):

"A comparison of surface electrostatics and hydrophobicity properties across the T4P species (Fig. S10 and Fig. S11) shows that all T4Ps feature a hydrophilic outer surface and a hydrophobic core. While the surface charge is predominantly negative or neutral, all T4Ps feature regions of positive charge of varying size (Fig. S10). As patches of positive charge may be related to the DNA binding capability of T4Ps reported in previous studies[54,55], the differences across species might indicate some variation in DNA binding capacity in the different species."

Minor comment: The EM density map, atomic model and validation reports should be available for the review process. The refinement statistics in the table are very good and the figures and supplementary movie present clear densities but this should be standard protocol and could help with constructive suggestions from the reviewers. Large map files, etc can be provided via a link if too big for upload directly through the manuscript tracking system.

The maps, models and validation are now available at the PDB (9EWX) and the EMDB (EMD-50025).

Reviewer #2 (Significance (Required)):

My main concern with this work is that it is quite minimalistic in terms of biology/physiology, especially in light of the many G- pili structures available, some of which the authors nicely review in terms of specific structural parameters. The hypothesis of increased protease or perhaps mechanical resistance is tantalizing but how the compact pilus fold actually affects Pseudomonas aeruginosa in its physiology is unclear. Are there any other differences in the surface properties relative to other pili (charge, surface motif conservation, etc.) and could they have relevance in terms of interactions with the substrate, another matrix component or a peculiar niche within the

host? As a PilA mutant should be easy to get from a number of laboratories, how would a Pseudomonas delta-PilA mutant behave in terms of twitching motility, surface attachment and biofilm formation if complemented with PaO1 PilA vs. other pilins from Table S2 or a pilin with an engineered hybrid architecture (e.g. a loop or b-hairpin insertion)? If such heterologous/engineered pili are incubated with a mild protease mix, would they indeed exhibit increased fragmentation relative to the wt PAO1 pili? To me, most of these assays are relatively easy to be attempted in terms of molecular biology, phenotypic assays and in vitro biochemistry (e.g. plasmid-based complementation if full genetics are judged beyond the scope/time available, twitching, pellicle formation, SDS-PAGE of protease-treated sheared pili) and could really shed more specific insights light into the peculiarities of Pseudomonas T4P function, rather than just present the next resolved filament among the multiple other T4P already out there.

While we agree with Reviewer #2 that the proposed experiments could provide further insights into *Pseudomonas* T4P function and would thus be of interest to perform, we feel that, as these experiments are extensive, they would be beyond the scope of this manuscript, as also pointed out by Reviewer #3. We believe that our high-resolution structure of this important biological molecule such as T4P will be of high value for further functional studies and will be particularly useful, as noted by Reviewer #1, in a drug discovery context.

Please also see our general comments at the beginning of this response document.

Reviewer #3 (Evidence, reproducibility and clarity (Required)):

In this manuscript Ochner et al. report the 3.2 Å cryo-EM structure of the type IV pilus (minus PilY1 adhesin) from P. aeruginosa PAO1. The authors demonstrate that the conserved N-terminal helix of pilin subunits (PilA) form a tubular arrangement within the hydrophobic core of the pilus whereas the divergent C-terminal pilin globular domain decorates the periphery of the pilus. Comparisons are then made against T4P structures from other organisms, highlighting interesting differences including a shorter rod diameter and lack of solvent-accessible loops for which the authors propose reduces proteolysis of the T4P compared to other organisms.

We thank Reviewer #3 for their comments and address their concerns point-by-point below.

Major comments:
The results of this manuscript are convincing. The models and cryo-EM volumes, which are already accessible from the PDB and EMDB, are of good quality with no obvious issues. The conclusions drawn from the model are not speculative. While extensive mutagenesis experiments could help delineate critical residues involved in T4P assembly and clarify involvement in adhesion/biofilm formation, these would have to be done in the native organism, would require a significant amount of time and effort, and would be beyond the scope of the current manuscript.

Minor comments:
The figures are excellent and clear, and the text is well-written, with results easy to interpret.

Thank you.

One of the strengths of this paper is the comparative analyses across current bacterial T4P structures. In this respect, I would have liked a more thorough analysis here:
• While differences in helical parameters, rod diameter, and rod length are presented, a figure showing comparison of surface electrostatics and/or hydrophobicity could help delineate differences (if any) across these species, which may reflect the different environments these bacteria inhabit.

We have added additional supplementary figures (Fig. S10, S11) comparing surface electrostatics and hydrophobicity across the species and commented on it in the main text (L202-208):

"A comparison of surface electrostatics and hydrophobicity properties across the T4P species (Fig. S10 and Fig. S11) shows that all T4Ps feature a hydrophilic outer surface and a hydrophobic core. While the surface charge is predominantly negative or neutral, all T4Ps feature regions of positive charge of varying size (Fig. S10). As patches of positive charge may be related to the DNA binding capability of T4Ps reported in previous studies[54,55], the differences across species might indicate some variation in DNA binding capacity in the different species."

• A consurf representation of PilA is shown in Fig. 3h. It would be helpful to include either the sequence alignment used for this analysis or a sequence alignment for all the species presented in the manuscript, to show precisely which residues are absolutely conserved across these species.
• A panel showing their full T4P as a surface with Consurf coloring would be informative to show conservation across the entire pilus and not just a PilA subunit.

We have added an additional Supplementary Figure (Fig. S8) showing both the pilin and the full pilus coloured by ConSurf conservation score as well as the sequence alignment used for the analysis. A file containing full sequence alignment information across all species will be provided as a Supplementary file (msa_fasta.aln).

• The authors state that the models in Fig. 3 were aligned based on the matchmaker function in Chimera. Wouldn't the poor sequence conservation of the C-terminal globular domain of PilA drive the alignment towards the N-terminal helix? In that case, wouldn't using a comparative alignment strategy that focuses on the model itself (LSQ) or secondary structure elements (SSM) which would drive the alignment more towards the globular domain be more reflective of the full pilin subunit?

The reviewer raises an interesting point here. Given the higher sequence conservation of this region, one might expect matchmaker to drive the alignment towards the N-terminal helix. However, due to the melted segment between the two helices in most of the pilin structures, alignment at the N-terminal helix would cause the globular domains not to be aligned, hence presumably the number of aligned residues is larger when aligning on the globular domain despite it being less conserved. As the alignment in this figure was mainly done for visualisation purposes, without drawing any conclusions about the conservation (which is highlighted by different colours in Fig. 3h specifically), this goal can be achieved using the alignment provided by the matchmaker function.

• Related to the point above, it would be useful to include a table highlighting pairwise RMSDs across all models presented in this manuscript.

We have included an additional table (Table S3) listing the RMSD values comparing the structure presented here to the T4P structures from other species and added a corresponding sentence to the main text (L183-185):

"To quantify these similarities and differences, we calculated RMSD values comparing the *P. aeruginosa* PAO1 model to all the other models presented here (Table S3)."

Reviewer #3 (Significance (Required)):

The authors rightfully highlight the importance of P. aeruginosa T4P in the development of biofilms; structural analyses of these pili are of clinical importance and of interest to researchers involved in bacterial motility.

To date, various structures of T4P and T4P subunits across a variety of bacterial have been solved by X-ray crystallography and cryo-EM (PDB: 9EWX (this study), 6GV9, 5VXX, 6XXD, 6VK9, 8TJ2). It appears that another group has recently published a slightly lower resolution (3.6 Å vs 3.2 Å) cryo-EM structure (PDB: 8TUM) of the T4P of P. aeruginosa PAO1 (Thongchol et al., Science, 2024). The model from this latter publication appears to be identical to the model presented in this manuscript. Since this work has now been published (with models being released mid-March 2024), and since Ochner et al.'s manuscript only appeared on Biorxiv on April 9th 2024, I feel it would have been appropriate and necessary to cite this paper. And while the Thongchol publication reduces the novelty of Ochner et al.'s manuscript, there is some merit in the comparative analyses performed, which if expanded upon, could further strengthen this manuscript enough to stand on its own.

We have included a citation of the paper by Thongchol et al. as requested (L218-222):

“We note that concurrent with the submission of this manuscript, a 3.6 Å-resolution cryo-EM structure of *P. aeruginosa* T4P, which largely agrees with our structural data, was reported in a study where the interaction of the T4P with single-stranded RNA phage PP7[68] was probed.”
