# Supplementary material for: Structure of the Pseudomonas aeruginosa PAO1 Type IV pilus
Source: PLoS Pathog. 2024 Dec 12;20(12):e1012773. doi: 10.1371/journal.ppat.1012773 (PMC11670995; doi:10.1371/journal.ppat.1012773)
Supplement: Attachment — Submitted filename: Ochner_ResponseToReviewers_R2.pdf [file ppat.1012773.s019.pdf]

Response to reviewers' comments for Ochner *et al*: PPATHOGENS-D-24-01538

Reviewer #1: The manuscript "Structure of the Pseudomonas aeruginosa PAO1 Type IV pilus" presents the high quality cryoEM reconstruction of the Type IV pilus (T4P) from an important human pathogen. This structure represents a clear advance from the previously published 8 Å T4P structure from P. aeruginosa strain K (PAK, ref. 26). The higher resolution allows the entire pilin chain to be fit. For the lower resolution structure reported previously only the N-terminal helix was resolved and the filament structure was obtained by fitting in the x-ray crystal structure of the PAK pilin subunit. Nonetheless, and despite the two pilins sharing only 67% identity, the two structures are highly similar, and this is never acknowledged in the current manuscript, which presents this T4P structure as completely new and novel. For instance, the abstract states that "PilA subunits constituting the T4P exhibit a classical pilin fold featuring an extended N-terminal a-helix linked to a C-terminal globular b-sheet-containing domain, which are packed tightly along the pilus. The N-terminal helices constitute the pilus core where they stabilise the tubular assembly via hydrophobic interactions. The a-helical core of the pilus is surrounded by the C-terminal globular domain of PilA that coats the outer surface of the pilus, mediating interactions with the surrounding environment." All of this was described for the lower resolution structure. In the first section of results (indeed the only results presented) the entire description they provide for the PAK pilus structure was described previously (ref. 26 for PAK pilus, and a multitude of papers for other T4P). The "key differences" and "common architectural principles" that this new structure apparently reveals are well-established. Thus, the findings are confirmatory, not novel, and while this high resolution structure is of value it's not clear what new information it provides beyond confirming the accuracy of the 8 Å structure. The manuscript should be reframed: present a side-by-side comparison of the two structures and highlight differences, if there are any.

We apologise if the impression the reviewer got was different, but we did not intend to claim that this is the first description of the Type 4 pilin architecture and have adjusted our manuscript extensively to make this point clearer. Briefly, we provide completely new figures 3 and S5 to highlight details revealed by our high-resolution structure, and also report a detailed comparison with previous models (see below).

We agree with the reviewer that the two pilins (PAK and PAO1) are sequence-related (reinforced in the new figure S5), and many architectural features of the PAK pilus, which are well-established in many other bacterial T4Ps, are also seen in the PAO1 pilus. This is now further reflected in the abstract and introductory texts.

Abstract:

"PilA subunits constituting the T4P exhibit a classical pilin fold featuring an extended N-terminal α-helix linked to a C-terminal globular β-sheet-containing domain, which are packed tightly along the pilus, in line with models derived from previous cryo-EM data of the *P. aeruginosa* PAK strain."

"Comparison of the *P. aeruginosa* PAO1 T4P with T4P structures from other organisms, both at the level of the pilin subunits and the fully assembled pili, confirms previously described common architectural principles whilst highlighting key differences between members of this abundant class of prokaryotic filaments."

Introduction:

"Previous studies have reported an X-ray crystal structure of the pilin monomer from *P. aeruginosa* strain K (PAK)[36], which was later integrated with an 8 Å resolution cryo-EM map of the *P. aeruginosa* PAK pilus[26] to propose a pseudo-atomic model of the PAK pilus. Despite this, a high-resolution structure of the filamentous T4P from *P. aeruginosa* has remained elusive, and there is little direct structural information available for the T4P of the highly studied model strain PAO1."

While relevant to our study, the PAK pilus is a different pilus, and given that PAO1 is one of the main strains of *P. aeruginosa* employed in a wide range of research, from twitching motility to biofilm formation, we believe that the structure of the PAO1 pilus is of value in its own right and that our structure is not just confirmatory of previous results. Moreover, as the reviewer states, a general advantage of having a high-resolution structure with side chain resolution is that it provides considerably higher certainty – allowing us to provide a detailed image of atomic interactions in Figures 2-3 which does not rely on docking of crystallographic data, and will as such be a significant resource for applications including drug discovery. To highlight the novel insights gleaned from our structural model, we have provided an additional Figure 3 pointing out some of the atomic interactions detected within our model along with a new paragraph in the main text.

"Moreover, ionic interactions and hydrogen bonds, mainly involving the loops in the pilin globular domains, stabilise the interfaces among multiple pilin subunits along the assembled pilus (Fig. 3). For example, the Q23 and Q25 residues of every pilin subunit N interact with the β3-β4-loop of subunit N-4 and the αβ-loop of subunit N-3 (Fig. 3c). Similarly, residue K139 of every subunit N interacts with the β2-β3-loop of subunit N+1 and the αβ-loop of subunit N-3 (Fig. 3d). These molecular bridges on the periphery together with the packed N-terminal helices in the centre result in an ordered, tubular pilus (Fig. 2a, 3a). While amino acid side chains were not resolved in previous cryo-EM reconstructions of the *P. aeruginosa* T4P, they can be resolved unambiguously in our map."

Please see the new figure 3 reproduced below for the reviewer's convenience.

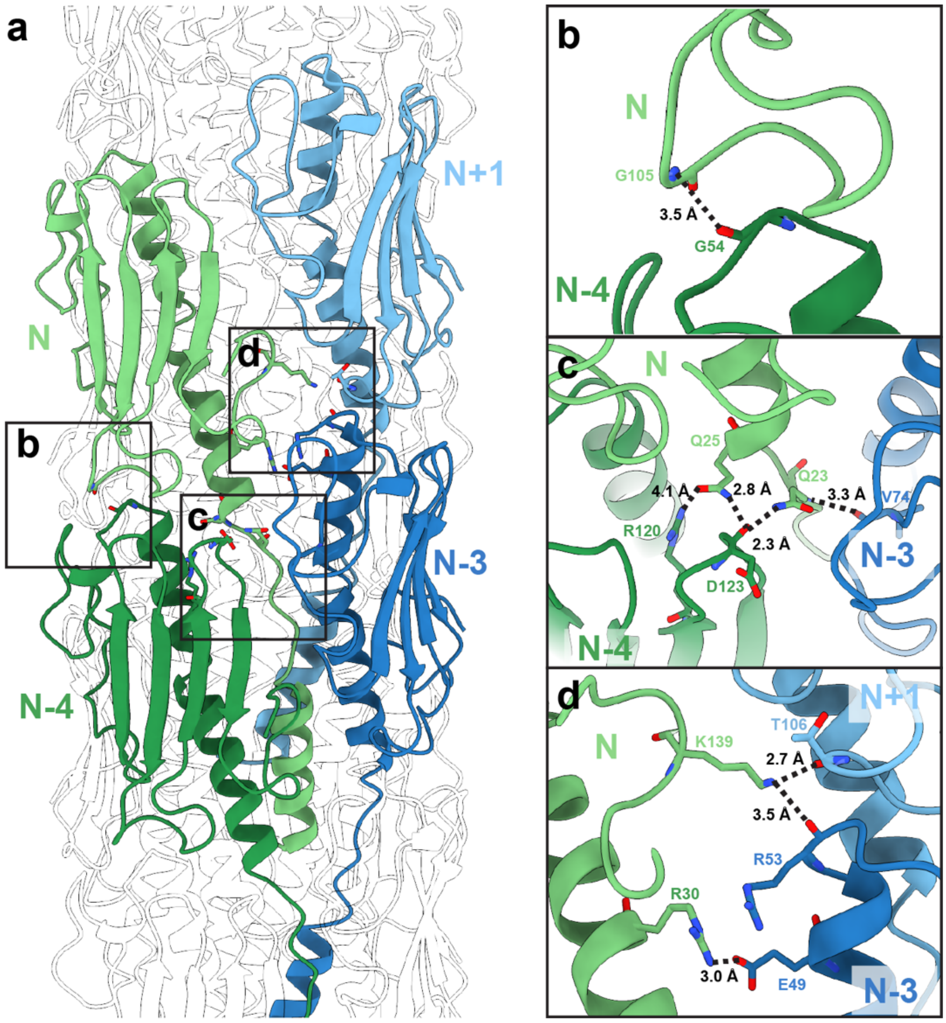

Figure 3: Interactions between pilin subunits at the periphery of the pilus.
a Structural model of four neighbouring pilin subunits (labelled N, N+1, N-3, N-4 based on the helical arrangement). **b** Interaction between the N and the N-4 subunits mediated through loops resolved in our 3.2 Å-resolution cryo-EM map. **c** Interactions at the interface among subunits N, N-3 and N-4 via side chains resolved in our cryo-EM map. **d** Interactions at the interface between subunits N, N-3 and N+1, via side chains resolved in our cryo-EM map.

To further acknowledge previous work, and to highlight differences, we agree with the reviewer's point that a more detailed comparison with the PAK pilus is warranted. We now provide a more detailed comparison of differing secondary structure elements within the pilin in Figure S5.

"In addition to the side chain information directly resolved in our 3.2 Å-resolution cryo-EM map, a direct comparison with the previously published model of the *P. aeruginosa* PAK strain pilus, which was derived from fitting an X-ray crystallographic model into an 8 Å-resolution cryo-EM map[26], shows differences even on the overall level of secondary structural elements (Fig. S5). The comparison highlights different relative orientations between the pilin globular domain and the N-terminal helix (Fig. S5c, d),

as well as significant differences in the size of αβ-loop (Fig. S5e). Furthermore, additional secondary structure formation within the loop region is observed (residues 56-58, 60-61, 72-73; Fig. S5e, marked by red asterisks), as well as differences in length of the β-strands, all of which are shorter in PAK than in PAO1, with the largest difference in the β4-strand (residues 126-130 (PAO1) vs residues 130-131 (PAK), Fig. S5f, marked by a red asterisk). These discrepancies between the two structural models likely originate from a combination of differences between the strains (Fig S5a), different experimental conditions between cryo-EM and crystallographic structural analysis, as well as the higher accuracy of the 3.2 Å-resolution cryo-EM map that we report in this study."

For the reviewer's convenience, we have added the new figure S5 below.

a

```
PAO1   FTLIELMIVVAIIGILAAIAIPQYQNYVARSEGASALATINPLKTTVEESLSRGIAGSKI  60
PAK    FTLIELMIVVAIIGILAAIAIPQYQNYVARSEGASALASVNPLKTTVEEALSRGWSV-KS  59
       **************************************::*********:**** :  *

PAO1   KIGTTASTATETYVGVEPDANKLGVIAVAIE-DSGAGDITFTFQTGTSSPKNATKVITLN  119
PAK    GTGTEDATKKEVPLGVAADANKLGTIALKPDPADGTADITLTFTMGGAGPKNKGKIITLT  119
         **  :* .*. :**  ******.**:  :  .*:.***:**  * :.***  *:***.

PAO1   RT-ADGVWACKSTQDPMFTPKGCDN     143
PAK    RTAADGLWKCTSDQDEQFIPKGCSR     144
       ** ***:* *.* **  * ****..
```

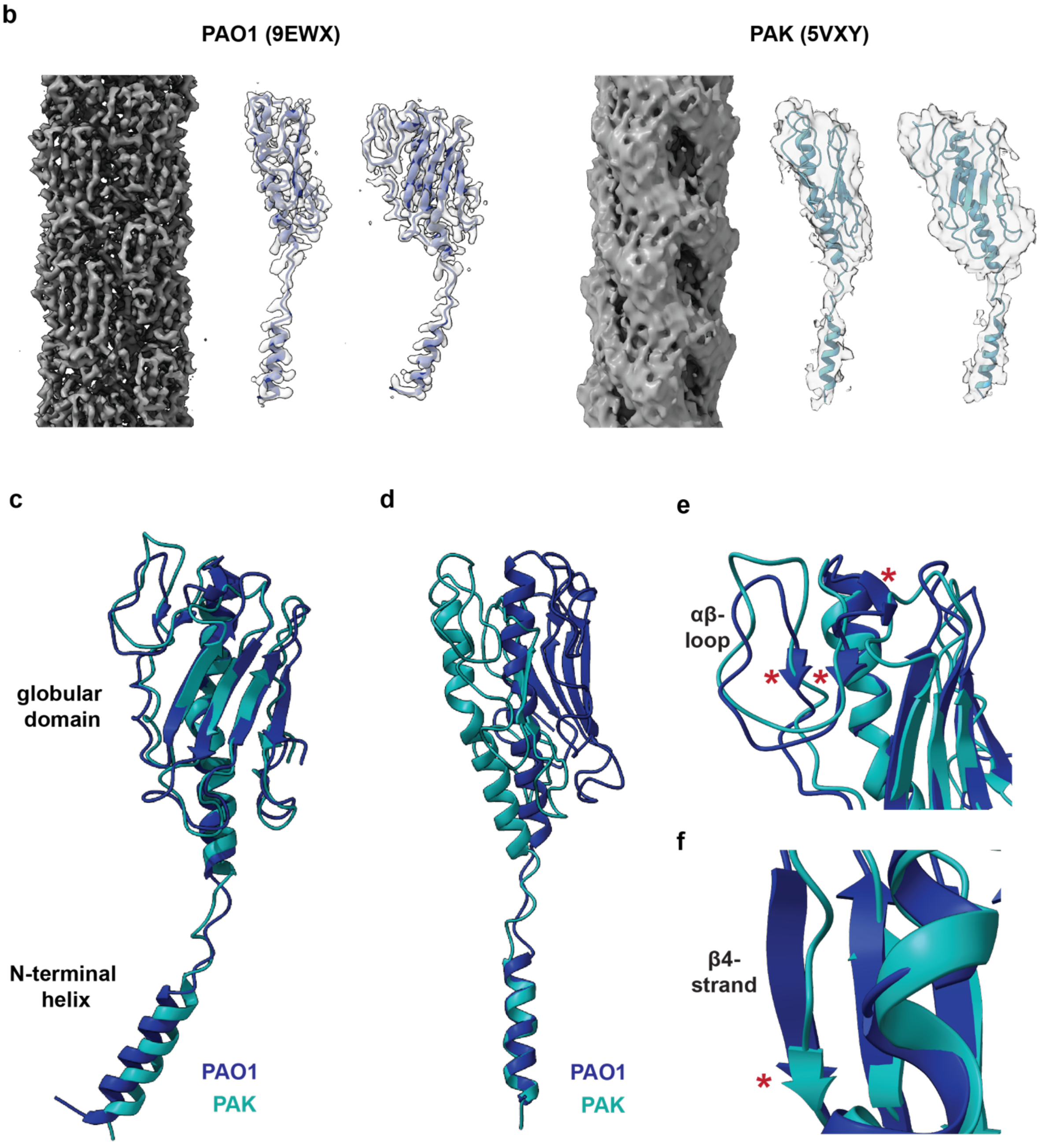

Figure S5: Comparison of the PAO1 T4P structure to the PAK T4P model.
a Sequence alignment[90] of *P. aeruginosa* strains PAO1 and PAK. **b** EM density map of the PAO1 T4P reported in this paper (EMD-50025), side view and front view of the EM density around one pilin subunit (PDB: 9EWX, this study) and EM density map of

the PAK T4P reported in a previous study[26] (EMD-8740), along with side view and front view of the EM density around one pilin subunit (PDB: 5VXY). **c-f** Alignment of the pilin structures of PAO1 (dark blue) and PAK (teal), based on the globular domain (**c, e, f**) or the N-terminal helix (**d**), shows secondary structure differences, highlighting the relevance of a high-resolution map featuring side chain densities. Specifically, there is a difference in orientation between the N-terminal helix and the globular domain (**c, d**). In the globular domain, PAO1 features additional small secondary structure elements within the αβ-loop, while the loop between beta strands β1 and β2 is extended in PAK (red asterisks) (**e**). There are also differences in beta strand length between the two structures (red asterisk) (**f**).

The authors provide some global comparisons of T4P structures and highlight some differences and speculate on their significance but this is a fairly superficial analysis. For example, in lines 177-180, they say that differences in the N-terminal a-helix position influence the pilus architecture but do not explain this statement. How do they influence the pilus architecture?

The relative positioning of N-terminal α-helix and the globular domain results in different helical parameters, which characterise pilus architecture. We have clarified this in the main text.

"While the globular domains are similar at an organizational level, the presence of the 'melted' linker segment results in a different positioning of the N-terminal helix relative to the globular domain, influencing the helical symmetry parameters and thus overall pilus architecture (Table S2)."

In lines 194-201 they speculate that the lack of surface-displayed loops makes P. aeruginosa T4P more resistant to proteolysis but provide no empirical data to support this.

We clarified further that this is a proposition, which would be an interesting line of further research.

"As flexible residues could potentially be targets for extracellular proteases, a lack of such residues might make the *P. aeruginosa* T4P more resistant to proteolysis, suggesting an interesting avenue of research to explore."

In line 205-208 they discuss the surface electrostatics and suggest that the positively charged regions may correlate with DNA binding regions but provide no analysis on which T4P are actually involved in natural transformation. (Note that the major pilin is not necessarily the site of DNA binding.)

We have clarified that DNA binding does not necessarily have to involve the major pilin.

We would like to note that we did not originally plan to include a discussion on DNA binding in the manuscript but have added this as a response to a request by a previous reviewer during the first round of revision.

"As patches of positive charge may be related to the DNA binding capability of T4Ps reported in previous studies[53,54], the differences across species might indicate some variation in DNA binding capacity in the different species; however, DNA binding mechanisms of T4Ps are not restricted to the major pilins, but can be mediated by other T4P components[55]."

Other points:

Line 107-151: The entire description of the T4P structure was described previously for the 8 Å PAK pilus structure. This section should start with a description of the map and compare it with 8 Å map to highlight the structural advance. Provide a sequence and structural comparison for the two T4P structures (and cite the recently published 3.6 Å PAK pilus structure, ref. 68), including of the pilin subunits. How do they differ? What is learned from this higher resolution structure.

We have added a new additional figure (Fig. 3) and a new paragraph to include more detail regarding side chain interactions to stress the additional level of information the higher resolution structure allows us to access.

We have also updated Fig. S5, which shows a comparison between the two maps to include further detail on secondary structure differences as well as a sequence comparison. The direct comparison reveals differences on the level of secondary structure, which can likely be traced to both differences between the protein sequence as well as differences arising from fitting the crystallographic model into a map vs direct modelling based on the high-resolution map.

We have cited and discussed the recently published 3.6 Å structure in discussion:

"We note that concurrent with the submission of this manuscript, a 3.6 Å-resolution cryo-EM structure of *P. aeruginosa* T4P, which largely agrees with and confirms our structural data, was reported in a study where the interaction of the T4P with single-stranded RNA phage PP7[68] was probed."

Line 113-114: "Next, we used a previous estimate of the helical symmetry of the P. aeruginosa
PAK strain T4P …". This was not an "estimate", it was an empirically determined value.

We have updated the text accordingly.

"Next, starting from previously reported helical symmetry parameters of the *P. aeruginosa* PAK strain T4P[26], whose PilA subunit has 67% sequence identity with the PAO1 PilA, we performed repeated three-dimensional classification and refinement (Fig. S2)."

Line 136: What is meant by “hydrophobic stacking”? This is a staggered arrangement, not a stacking.

We agree and have updated the text to reflect the staggered arrangement.

“The innermost part of the T4P is stabilised by hydrophobic interactions of the N-terminal α-helices of PilA, which contain a row of hydrophobic residues (amino acid residues 1-16, where residue 1 is the N-terminus of the mature pilin), forming the core of the T4P in a staggered arrangement (Fig. 2d).”

Line138: “residues 7-22” Residues are numbered based on the pre-pilin. The convention for T4 pilins is to number the residues beginning at the mature protein. Thus, this should be “residues 1-16”.

We have updated the numbering accordingly in the manuscript text as well as in the figures.

Reviewer #2: This is a revised submission of a previously reviewed paper on the 3.2 Å cryo EM structure of the P. aeruginosa PAO1 type IV pilin structure. The structure is well-executed and the experimental methodology is sufficiently explained. Although there is a contemporaneous publication of a 3.6 Å structure of the same filament, this is still a useful publication at slightly higher resolution that verifies and expands those results.

The authors seem to have made significant changes in response to the previous review.

We thank the reviewer for their positive comments.

Part III – Minor Issues: Editorial and Data Presentation Modifications
Please use this section for editorial suggestions as well as relatively minor modifications of existing data that would enhance clarity.

Reviewer #1: (No Response)
Reviewer #2: I have only one comment to address.
1. On page 163, and in Fig. S9, the crystal and EM structures are compared and the “melted” region in the EM studies is noted as a difference. Authors should clarify it’s not more or less accurate to have the unraveled helix than the helical section in the crystal structures, and it is not the case that different bacterial pilins would have different structures here, nor that the resolution plays any role in the differences. Rather, the crystal structures are determined of pilin subunits in biological detergents, and the alpha-1 helices in these cases have relaxed to the lowest free

energy structure, the helix. On the other hand, in assembled filaments, the unraveled helix is maintained.

We thank the reviewer for their comment and have clarified this in the manuscript.

"While in X-ray structures of pilins, including that of the *P. aeruginosa* PAK strain, the experimental conditions lead to a relaxation of the N-terminal part of the pilin into a continuous helix spanning the full length of the subunit[49,50] (Fig. S9), many cryo-EM structures of pilins determined within the full pilus architecture– including our 3.2 Å-resolution structure – feature a non-helical but well-resolved linker segment (Fig. 2c, S4b) containing a prominent helix-breaking proline residue (P22, Fig. S4b), which divides the α-helical part of PilA into an N-terminal helix (residues 1-16) and a distinct α-helix that is part of the globular domain (residues 25-52)."
